# Development and in vitro validation of flexible intraretinal probes

**DOI:** 10.1038/s41598-020-76582-5

**Published:** 2020-11-16

**Authors:** V. Rincón Montes, J. Gehlen, S. Ingebrandt, W. Mokwa, P. Walter, F. Müller, A. Offenhäusser

**Affiliations:** 1 Bioelectronics, Institute of Biological Information Processing-3, Forschungszentrum Jülich, Jülich, Germany; 2 Molecular and Cellular Physiology, Institute of Biological Information Processing-1, Forschungszentrum Jülich, Jülich, Germany; 3 Institute of Materials in Electrical Engineering 1, RWTH Aachen University, Aachen, Germany; 4 Department of Ophthalmology, RWTH Aachen University, Aachen, Germany; 5 RWTH Aachen University, Aachen, Germany

**Keywords:** Eye diseases, Electrophysiology

## Abstract

The efforts to improve the treatment efficacy in blind patients with retinal degenerative diseases would greatly benefit from retinal activity feedback, which is lacking in current retinal implants. While the door for a bidirectional communication device that stimulates and records intraretinally has been opened by the recent use of silicon-based penetrating probes, the biological impact induced by the insertion of such rigid devices is still unknown. Here, we developed for the first time, flexible intraretinal probes and validated in vitro the acute biological insertion impact in mouse retinae compared to standard silicon-based probes. Our results show that probes based on flexible materials, such as polyimide and parylene-C, in combination with a narrow shank design 50 µm wide and 7 µm thick, and the use of insertion speeds as high as 187.5 µm/s will successfully penetrate the retina, reduce the footprint of the insertion to roughly 2 times the cross-section of the probe, and induce low dead cell counts, while keeping the vitality of the tissue and recording the neural activity at different depths.

## Introduction

Worldwide, the third leading cause of blindness is retinal degenerative diseases caused by photoreceptor loss, such as age-related macular degeneration (AMD) and retinitis pigmentosa (RP). Efforts to treat blind patients with AMD and RP go from the regulation of vitamins, to the development of novel experimental strategies like stem-cell based therapy, gene therapy, transplantation of lost retinal tissue, and visual prostheses^1–3^. To restore the lost function of photoreceptors while using the remaining healthy network in the retina, visual prostheses that stimulate the retina electrically are used. Such devices contain mainly a light sensitive and transduction unit (e.g. a camera and pulse generator or a photodiode array), a signal processing unit, and a microelectrode array (MEA) that interfaces directly with the retina and delivers the electrical stimuli^4^.

To date, retinal implants have shown the restoration of useful vision in blind patients^5^. However, current retinal implants do not provide feedback on the ongoing retinal activity and the efficiency of the stimulation and do not adjust to the remodelling processes within the degenerated retina^6–9^. Thus, the development of penetrating silicon (Si) bidirectional microelectrode arrays (BiMEAs) for retinal applications has opened a new door to access different retinal layers, thereby allowing access to electrically stimulate the residual inner retina while monitoring not only the local field potentials (LFPs) and spontaneous spiking activity, but also the responses of the remaining retinal network to electrical and/or optical stimulation. Nonetheless, the biological impact on the retina induced by the insertion of such rigid devices is still unknown^9^.

To further develop a bidirectional communication strategy using penetrating BiMEAs^8,9^ in retinal applications, and given the stiffness of Si-based implants^10,11^, the goal of this work is to reduce and evaluate the acute biological impact of intraretinal devices. To this end, BiMEAs based on flexible materials were developed and tested for the first time in vitro in wildtype and degenerated mouse retinae. Furthermore, an assessment of the acute insertion impact in healthy mouse retinae was performed in comparison with the standard Si-BiMEAs. The study presented here exposes the design principles necessary to meet the insertion requirements of flexible intraretinal probes. It reveals that flexible BiMEAs are able to penetrate the retina, while keeping the vitality of the tissue and reducing the acute insertion footprint, thereby proving the feasibility of using such devices for in vivo retinal applications.

## Results

### Development of flexible intraretinal probes

**Figure 1:**
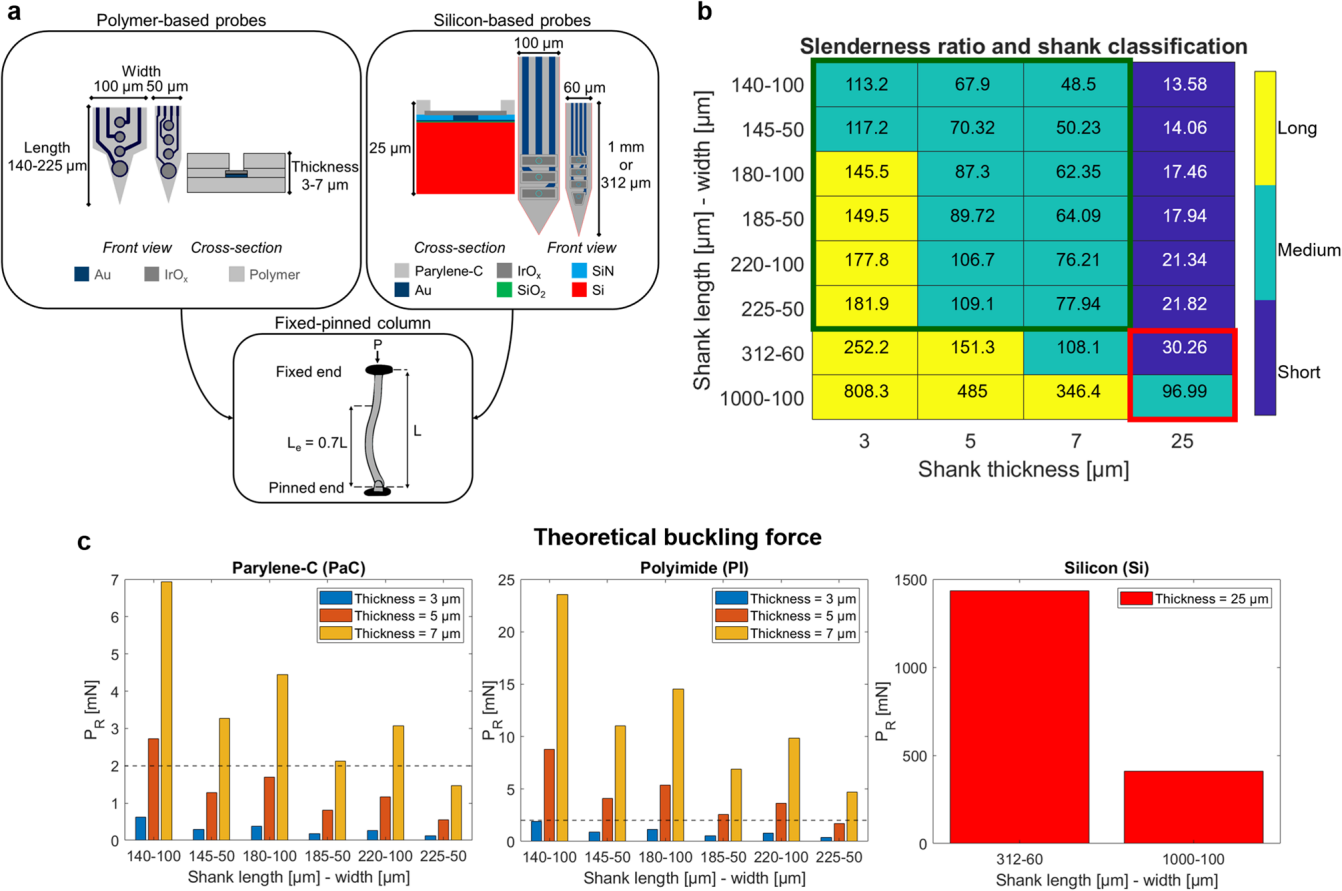
Intraretinal insertion model. (**a**) Schematic and dimensions for the flexible (left box) and silicon (right box) intraretinal probes, which are assumed as fixed-pinned columns (lower box) in the insertion model. L depicts the initial length and L_e_ stands for the effective length of the column*.* (**b**) Slenderness ratio (λ) and colour-coded shank classification as long (yellow), medium (aquamarine), and short (dark blue). λ for the dimensions of interest and for the dimensions of the silicon shafts are enclosed in a green and red square, respectively. (**c**) Theoretical buckling force (P_R_) of the proposed dimensions for parylene-C (left box) and polyimide (middle box) intraretinal shanks with thicknesses of 3 µm (blue bars), 5 µm (orange bars), and 7 µm (yellow bar). The dashed line depicts an insertion force threshold of 2 mN. Additionally, P_R_ of silicon shanks with a thickness of 25 µm (red bar) is shown in the right box as reference.

Intraretinal probes as proposed by the BiMEA consortium^9,12^ consist of penetrating multi-shank and multi-site MEAs, made up initially of Si-based substrates. Having as reference the development of cortical and intracortical implants^13–18^, flexible BiMEAs were designed and fabricated to better match the anatomy and mechanical properties of the retina. To this end, the following strategy was pursued: (i) Considering that the probes were to be tested in mouse retinae, the length of the penetrating shafts (140–225 µm) was first selected to span the thickness of the target tissue, which can be ~ 200–220 µm in healthy mice^19^ and ~ 100–120 µm in mice with degenerated retinae^20,21^. (ii) As illustrated in Fig. 1a, the dimensions of flexible BiMEAs have a reduced cross-section compared to Si-BiMEAs from 1500–2500 µm^2^ (widths of 60 or 100 µm and a thickness of 25 µm) to 150–700 µm^2^ (widths of 50 or 100 µm and a thickness of between 3 and 7 µm), thereby minimizing the cross-sectional footprint of the shanks 3.5–10 times. (iii) Due to the known stiffness of Si (hundreds of GPa), biocompatible and flexible materials, such as polyimide (PI) and parylene-C (PaC), with a Young’s modulus lower than tens of GPa^13–15^ were selected for the development of flexible intraretinal probes.

In light of the challenge reported by different research groups to insert flexible intracortical implants into the brain^13–15,22^, the feasibility of inserting flexible shanks into the retina was investigated using an insertion model based on the computation of the theoretical critical buckling load^13,23,24^ and performing insertion tests into a retina phantom. First, both flexible (polymer-based) and Si-based shanks were assumed as fixed-pinned columns (Fig. 1a), as shanks are fixed from the top by the holder-contact pad-board complex (Supplementary Figure 1), and pinned from the tip, which initially anchors the shank to the tissue. To determine whether Euler’s or Rankine-Gordon’s buckling load formula should be used to compute the buckling force of a column, the slenderness ratio (λ) was calculated, which relates in turn to the theoretical capability of a shank to buckle^25^.

Given the above, shanks were first classified as long (λ > 120), medium (32 ≤ λ ≤ 120), or short columns (λ < 32)^25,26^ as shown in Fig. 1b. For the dimensions of interest (enclosed with a green square), flexible shanks fall in the category of long columns when selecting shanks with lengths between 180 and 225 µm and a thickness of 3 µm, or medium columns if the length is 140-145 µm and the thickness is 3 µm or all lengths of interest with 5–7 µm thickness. On the other hand, with a thickness of 25 µm the dimensions of Si-BiMEAs represent short and medium columns at lengths of 312 µm and 1000 µm, respectively (see red square). Hence, long, narrow, and thin dimensions increase the potential for a shank to bend (higher values of λ), while short, wide, and thick dimensions will reduce buckling (lower values of λ).

Euler’s formula is commonly used to assess intracortical shafts and applies only for long columns in the computation of the buckling force^25,26^. Considering that the shaft dimensions required for our design correspond to middle and long columns, Rankine-Gordon’s buckling load formula (P_R_) was employed. Thus, the theoretical P_R_ is displayed in Fig. 1c for PaC and PI and for Si as reference. In order to avoid failure due to bending during the insertion of penetrating probes, the insertion force (F_ins_) into the tissue should be lower than the P_R_ of the shank^15^.

Considering that the insertion forces to penetrate the retina were not found in the literature, that the mechanical properties reported by different research groups suggest that the retina might be as soft as the brain or slightly stiffer than the brain^27–31^, and that intracortical insertion forces range between 0.5 to 2 mN^13^, a conservative insertion force threshold of 2 mN was used to assess theoretically the feasibility of inserting flexible intraretinal probes. Therefore, shanks with a thickness of 7 µm, a length between 140 and 220 µm, and a width of 50 or 100 µm will theoretically penetrate the target tissue for both flexible materials. For a thickness of 5 µm, only the shortest and widest PaC shanks, as well as PI shanks with all the dimensions of interest (except 225 µm in length) will also be able to insert into the tissue without bending. On the contrary, the P_R_ of 3 µm thick flexible shanks is lower than 2 mN, indicating that these will fail due to bending during insertion if F_ins_ is higher than P_R_. As for the Si case, the longest shank with a thickness of 25 µm presents the lowest P_R_ among the two reference dimensions, which is 17 and 59 times higher than the highest P_R_ of PaC and PI columns, respectively. Thus, results of Si-BiMEAs reported previously support the model, as intraretinal insertions of Si shanks were accomplished successfully without bending^9^.

From theory to practice, dummy flexible BiMEAs were used to test the insertion of shanks presenting the lowest P_R_ for each width proposed (length–width of 225–50 µm and 220–100 µm). Insertion tests were conducted into a polydimethylsiloxane (PDMS) phantom tissue that mimicked an average elastic modulus of the retina, ~ 29 kPa (Supplementary Figure 2). As was predicted from the calculation of P_R_, 7 µm thick PaC and PI shanks were successfully inserted into the phantom, as well as 5 µm thick PI. Surpassing our expectations, positive results were also obtained for 5 µm thick PaC and even 3 µm thick PI shanks, thereby suggesting that the insertion forces were lower than the critical buckling load of the samples. Unfortunately, 3 µm thick PaC shanks were not tested as their final assembly failed due to rolling of the contact pad area after its release from the wafer. Moreover, dimpling was observed (white arrows in Supplementary Figure 2), indicating that compression of the target tissue would be expected during in vitro insertions.

Given the above, the insertion feasibility of intraretinal shanks was demonstrated, and even more, results revealed that the probes could be used without the need of an insertion aid. Considering that the insertion forces were not measured, flexible shanks with a thickness of 7 µm were chosen for further in vitro experiments because they provide the highest buckling force threshold among the optimised dimensions.

### In vitro recordings with flexible intraretinal probes

Once the shank tips were placed at the epiretinal surface of the prepared retinal tissue, stepwise insertions with a continuous insertion force during each step were carried out. Thus, the insertion of flexible BiMEAs carrying iridium oxide electrodes (IrO_x_) with a diameter of 15 µm (upper electrodes) and 25 µm (lower electrodes) was accomplished performing one or two long insertion step(s) (Z_in_) between 40 and 180 µm. In this way, the puncture of the first retinal layer was induced (see Complementary video 1), which was confirmed by the recording of action potentials (APs) at the lower electrodes of the shanks. When necessary, subsequent insertion steps (ΔZ) between 20 and 40 µm were performed to drive the shanks to the desired intraretinal depths.

**Figure 2:**
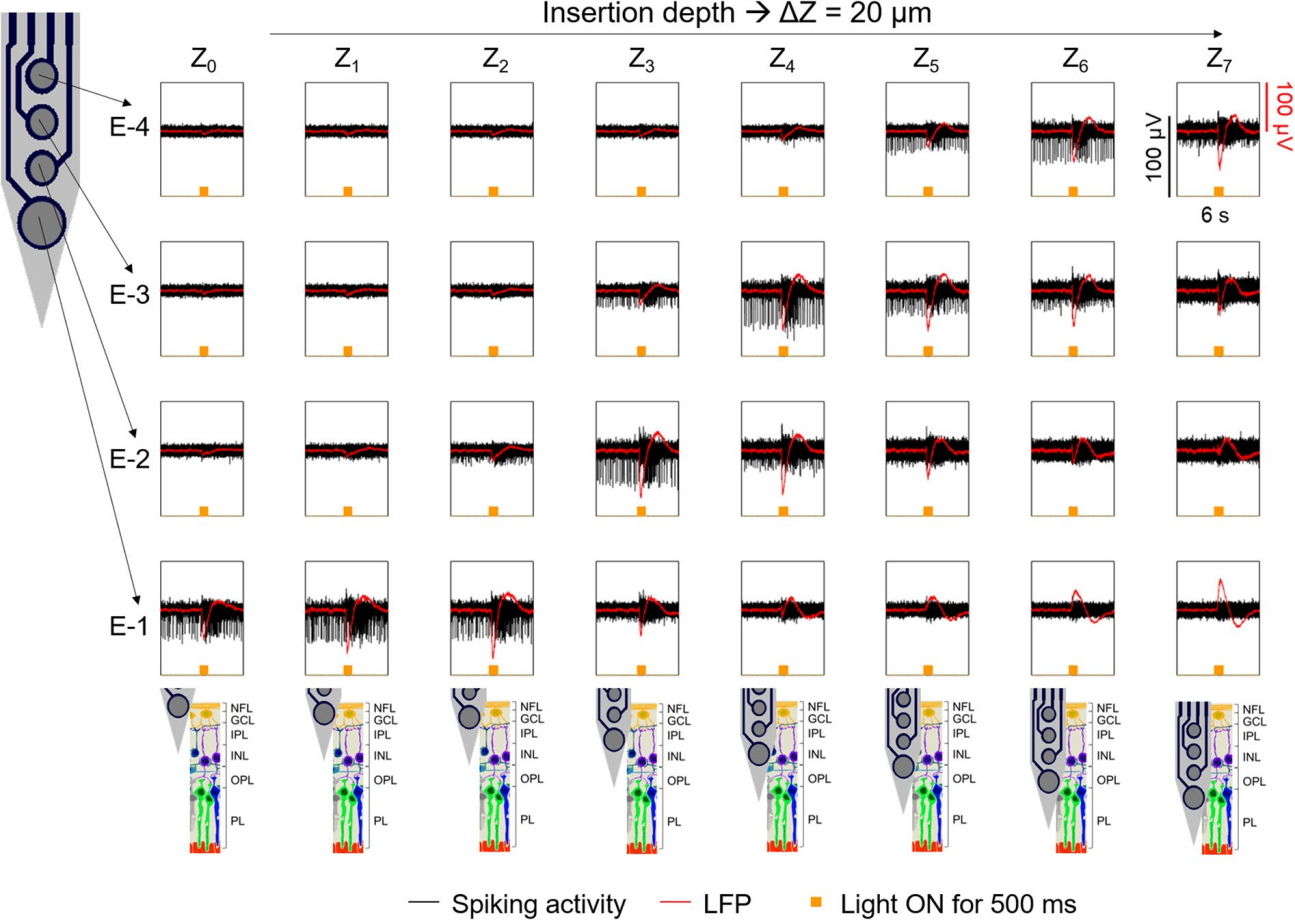
Recordings during the intraretinal insertion of flexible BiMEAs in wildtype mouse retina. Recording snapshots of the intraretinal activity during insertion of a flexible PI shank (50 µm wide, 185 µm long) are displayed for every electrode along the shank (rows) and every insertion depth Z_x_ inside the retina (columns). Z_in_ used a speed of 162.5 µm/s. After the initial insertion step of 180 µm, Z_0_ was set as the retinal surface. Further insertion steps, ΔZ, of 20 µm were carried out to position the electrodes at different retinal layers. The spiking activity of the retina and local field potentials (LFP) are shown in black and red, respectively, while performing light stimulation of the tissue with a stimulus 500 ms long (yellow bumps). Illustrations of the expected position of the electrodes inside the retina are shown at the bottom, where intraretinal layers are coded as: NFL = nerve fibre layer, GCL = ganglion cell layer, IPL = inner plexiform layer, INL = inner nuclear layer, OPL = outer plexiform layer, and PL = photoreceptor layer.

An example of an intraretinal insertion with a flexible PI probe in a wildtype (WT) mouse retina using an insertion speed (V_in_) of 162.5 µm/s, a Z_in_ of 180 µm, and subsequent ΔZ steps of 20 µm is displayed in Fig. 2. After Z_in_ was performed, spikes were captured at the lower electrode, thereby indicating that the nerve fibre layer (NFL), referred to as the surface of the retina (Z_0_), was reached. As multiple ΔZ were carried out, the detected spiking activity of retinal ganglion cells (RGCs) was shifted from the bottom to the upper electrodes while the shank was driven deeper inside the retina. Hence, the displacement of the signal is a clear indication that the shanks crossed the retinal layers, which agrees with findings reported previously^9^.

Additionally, a 500 ms long light stimulus was applied at every depth to check the vitality of the tissue. Vitality was confirmed by the bursting responses and LFPs captured during optical stimulation throughout the intraretinal insertion. Moreover, LFPs, which resembled the waveform of an intraretinal electroretinogram (ERG), exhibited a reversed polarity behaviour as the electrodes moved into the inner retina (E-1 at Z_4_–Z_5_), a behaviour that matches reports by Tomita and Funaishi^32^ after recording intraretinal ERGs using a glass micropipette with a silver microwire. Previous recordings performed with Si exhibited light induced artifacts at the onset and offset of the stimulus due to photoelectric effects, which are not visible in polymer-based probes. Therefore, this is the first time that intraretinal ERG-like waveforms are captured intraretinally with multi-site penetrating shanks.

**Figure 3:**
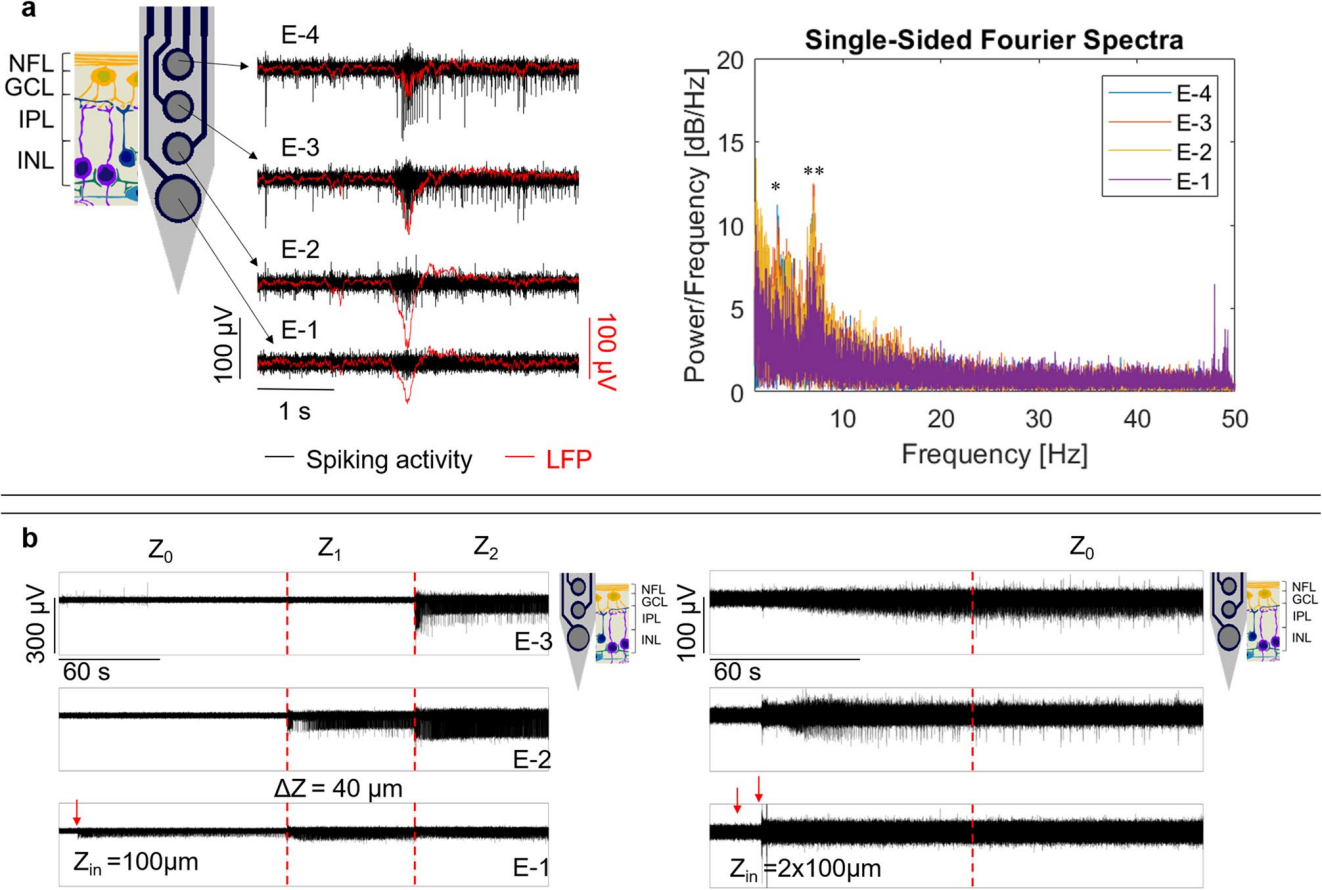
Recording the intraretinal insertion of flexible BiMEAs in *rd10* mouse retina. (**a**) At the left, the electrical activity captured by a fully inserted PaC shank (50 µm wide and 185 µm long) is shown. The spiking activity and the local field potentials (LFPs) are exhibited in black and red, respectively, for the electrodes of one intraretinal shank. At the right, the corresponding single-sided Fourier spectrum is exhibited for each electrode, presenting peak frequencies at 3.3 Hz (*) and 7 Hz (**). (**b**) Merged recordings of the spiking activity captured throughout different insertion steps (Z_0−x_) by a PaC probe (50 µm wide and 145 µm long). Left: An initial insertion step (Z_in_) of 100 µm and further steps (ΔZ) of 40 µm were used. Right: A Z_in_ equivalent to two steps of 100 µm with a lag of 10 s was performed. For both plots, each row corresponds to one of the three electrodes along each shank, with E-1 and E-3 the lower and upper electrodes, respectively. Red dashed lines separate different insertion depths, and the red arrows point out when Z_in_ was performed. For (**a**) and (**b**), an insertion speed of 187.5 µm/s was used. Additionally, schematics illustrating the expected insertion depth of the shanks are shown, where intraretinal layers are coded as: NFL = nerve fibre layer, GCL = ganglion cell layer, IPL = inner plexiform layer, INL = inner nuclear layer.

Furthermore, intraretinal insertions performed in *rd10* retinae allowed the recording of pathologic activity present in degenerated mouse retinae^7,33–35^, showing a burst of spikes concomitant with low frequency oscillations (Fig. 3a, left) ranging between 3 and 7 Hz (Fig. 3a, right). This behaviour is consistent with intraretinal recordings reported previously using Si-based devices^9^. Moreover, flexible shanks as short as 145 µm were successfully inserted within the thin degenerated retina (Fig. 3b). Likewise, after using a higher V_in_ (187.5 µm/s), longer insertion steps after Z_in_ (e.g. ΔZ = 40 µm in Fig. 3b, left), and performing two consecutive insertion steps of 100 µm with a lag of ~ 10 s (Fig. 3b, right), it was observed that the number of insertion steps required to penetrate the tissue were dependent on the length of Z_in_, the size of further steps after Z_in_, and V_in_. Hence, depending on the purpose of the experiment, Z_in_, ΔZ, and V_in_ can be adjusted to capture the electrical behaviour of the retina at different intraretinal depths.

### Acute insertion impact of intraretinal probes

The acute insertion trauma of intraretinal probes was assessed by determining the insertion trauma area (ITA), defined as the area comprising the insertion hole and the dead cells around or within the hole, the insertion trauma area ratio (ITR), defined as the ratio of ITA to the shank cross-section, and the number of dead cells encountered in ITA, in the following referred as count of dead cells. Likewise, the main substrate material (Si, PI, or PaC), the cross-section (350 or 700 µm^2^ for flexible probes and 1500 or 2500 µm^2^ for Si probes), and V_in_ (62.5, 112.5, 162.5, or 187.5 µm/s) were considered as independent parameters affecting the acute insertion trauma of intraretinal probes. According to the material and shank width of the tested samples, they will be referred hereafter as Si-100, Si-60, PI-100, PI-50, PaC-100, and PaC-50.

**Figure 4:**
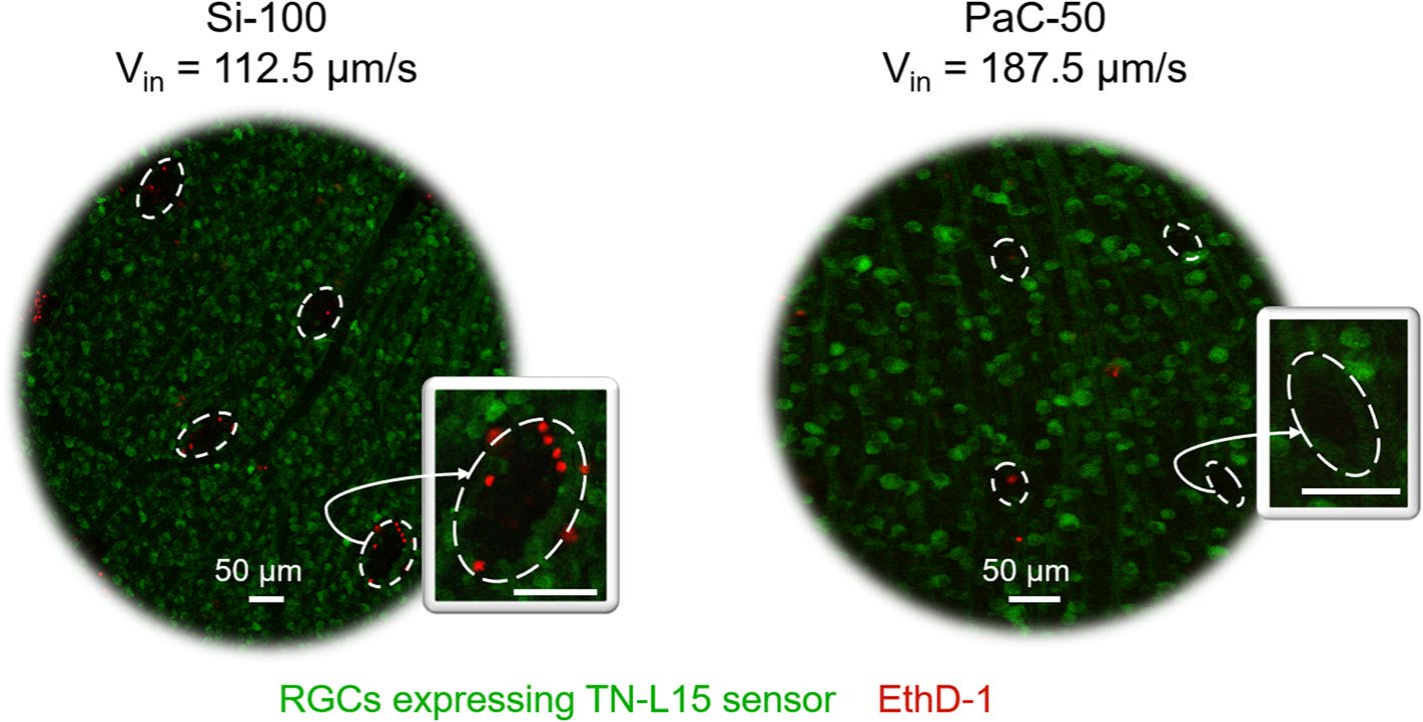
Dead cell stainings after intraretinal insertions in TN-L15 mouse retinae. Ethidium homodimer (EthD-1) was used to stain dead cells (red) in TN-L15 retinae containing RGCs expressing the Ca^2+^ sensor TN-L15 (green). The images show the top view of the maximum intensity projection of stainings that exhibited one of the biggest (left) and smallest (right) insertion trauma areas (ITAs), which are enclosed with white dashed lines.

Two examples displaying the staining of dead cells (red) after intraretinal insertions in transgenic TN-L15 mouse retinae, which were chosen based on their strong visible fluorescence in RGCs (green), are shown in Fig. 4. Stainings for each tested condition can be found in Supplementary Figure 3. As an overall picture, it can be observed that the ITA (enclosed with white dashed lines) caused by Si-100 (Fig. 4, left) was bigger than for the rest of the samples. Likewise, ITA was smaller and fewer dead cells were counted when probes with narrow shanks and a high V_in_ were used, as for Si-60, PI-50, and PaC-50 using a V_in_ of 187.5 µm/s (Fig. 4, right).

**Figure 5:**
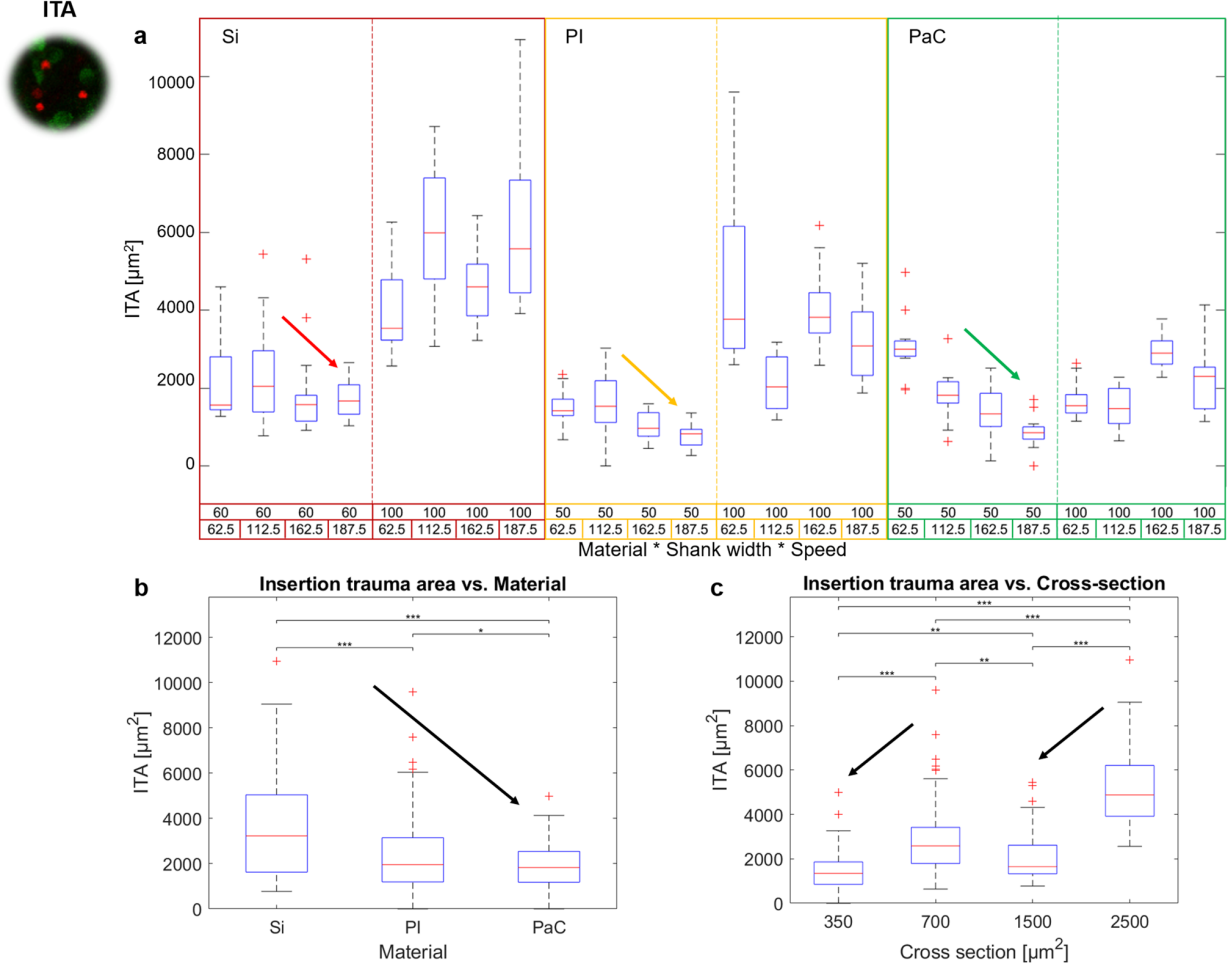
Insertion trauma area after acute intraretinal insertions. The effects of the probe material, the shank cross-section, and the insertion speed on the insertion trauma area (ITA) are shown. (**a**) Overall boxplot comparison among the probes grouped by material, shank width, and insertion speed. (**b**) Boxplot comparison among materials. (**c**) Boxplot comparison among different shank cross-sections. Cross-sections corresponding to 350 and 1500 µm^2^ correspond to shank widths of 50–60 µm, while 700 and 2500 µm^2^ depict shank widths of 100 µm. Additionally, PI and PaC are linked to cross sections of 350 and 700 µm^2^, while Si is linked to 1500 and 2500 µm^2^. Arrows show the main trends. Significant differences in (**b**) and (**c**) are shown after performing post-hoc pairwise testing using non-parametric bootstrap t-tests and Bonferroni correction (* for *p* ≤ 0.05, ** for *p* ≤ 0.01 *** for *p* ≤ 0.001).

Furthermore, the comparison of ITA among the different probes grouped by material, shank width, and V_in_ revealed that the combination of using PI or PaC probes with narrow shanks and a V_in_ of 187.5 µm/s generated the smallest ITA with an average size of 782.17 ± 309.48 and 861.7 ± 396.72 µm^2^, respectively (Fig. 5a). These results were significantly lower than 70% of the overall samples, having a lower impact than all insertions performed with Si-100 and PI-100, lower than at least three out of four insertion conditions tested for Si-60 and PaC-100, and lower than PI-50 and PaC-50 when low insertion speeds were used (Fig. 5a and Supplementary Figure 4a).

Although Si and flexible probes had different thicknesses (Si was 25 µm and polymers were 7 µm thick), a significant influence of the material was detectable (Fig. 5b), since PaC caused significantly smaller ITA compared to both Si and PI by an average factor of ~ 1.2–1.9 times lower. Likewise, differences in the effects of shank cross-sections were highly influenced by the width, as those corresponding to the widest shanks (700 and 2500 µm^2^) produced an overall ITA that was ~ 2–2.5 times higher than widths between 50 and 60 µm (Fig. 5c). Whereas no clear trend was identified for each material over different V_in_ (Supplementary Figure 4b), ITA was significantly reduced in narrow shanks when increasing V_in_ (Supplementary Figure 4c), a trend that is also visible in Fig. 5a.

**Figure 6:**
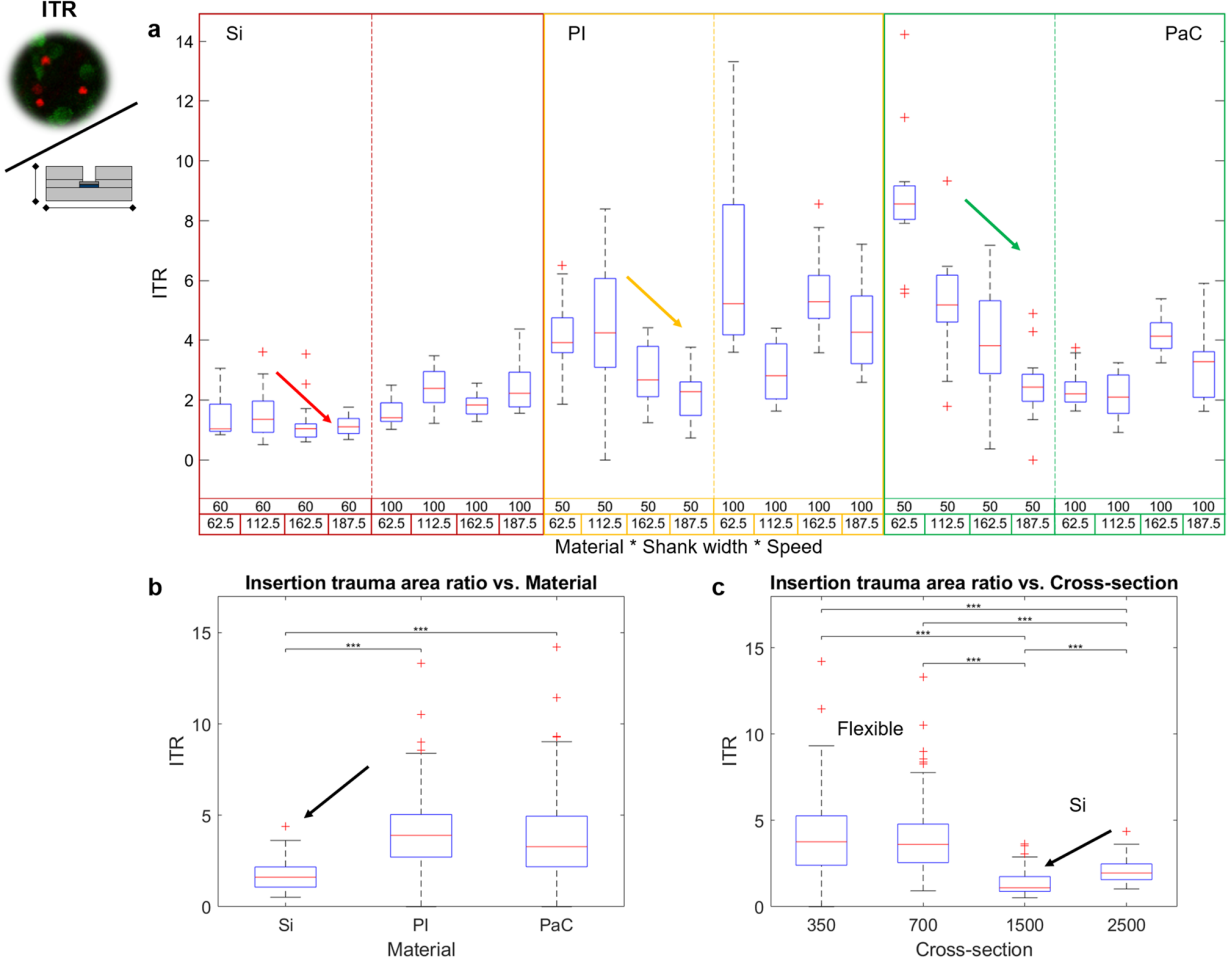
Insertion trauma area ratio of intraretinal probes. The effects of the probe material, the shank cross-section, and the insertion speed on the insertion trauma area ratio (ITR) are shown. (**a**) Overall boxplot comparison among the probes grouped by material, shank width, and insertion speed. (**b**) Boxplot comparison among materials. (**c**) Boxplot comparison among different shank cross-sections. Cross-sections corresponding to 350 and 1500 µm^2^ correspond to shank widths of 50–60 µm, while 700 and 2500 µm^2^ depict shank widths of 100 µm. Additionally, PI and PaC are linked to cross sections of 350 and 700 µm^2^, while Si is linked to 1500 and 2500 µm^2^. Arrows show the main trends. Significant differences in (**b**) and (**c**) are shown after performing post-hoc pairwise testing using non-parametric bootstrap t-tests and Bonferroni correction (* for *p* ≤ 0.05, ** for *p* ≤ 0.01 *** for *p* ≤ 0.001).

On the other hand, Si-probes plainly showed the lowest ITR compared to both PI and PaC, indicating that the ITA in Si was closer to the actual shank cross-section (Fig. 6a,b and Supplementary Figure 5a,b). This trend was also observed when the shank cross-sections were compared, as those corresponding to Si yielded the lowest ITRs (Fig. 6c). In addition, a significant difference between Si-60 and Si-100 reflected a reduced ITR for smaller shank cross-sections in Si devices. For Si, proportionality of the cross-sectional area to the ITA agrees with results reported for intracortical applications with Si devices, in which the initial insertion footprint is proportional to the cross-section of the device^36^, however, as revealed by results in Fig. 6, this premise is not true for flexible materials like PI and PaC. The latter could be a result of surface differences among materials^37^, for example the hydrophobicity of PaC.

Reviewing the literature, it can be observed that in general, the normalisation of the ITA to the cross-section of the device is bigger for flexible than for Si probes (Supplementary Table 1), however, the latter has not influenced the fact that flexible neural probes have reduced foreign body reactions in chronic applications when compared to Si^10,11^. Nonetheless, high ITRs of flexible shanks was compensated when increasing V_in_ in narrow shanks, a trend that was also valid for Si (Fig. 6a and Supplementary Figure 5c). Consequently, the lowest ITRs were obtained for Si-60 (1.17 ± 0.34), PI-50 (2.17 ± 0.86), and PaC-50 (2.46 ± 1.13) when a V_in_ of 187.5 µm/s was used.

**Figure 7:**
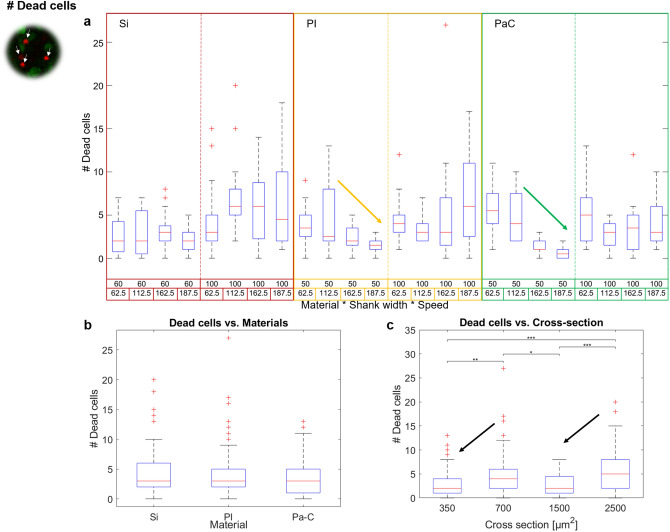
Count of dead cells after acute intraretinal insertions. The effects of the probe material, the shank cross-section, and the insertion speed on the count of dead cells are shown. (**a**) Overall boxplot comparison among the probes grouped by material, shank width, and insertion speed. (**b**) Boxplot comparison among materials. (**c**) Boxplot comparison among different shank cross-sections. Cross-sections corresponding to 350 and 1500 µm^2^ correspond to shank widths of 50–60 µm, while 700 and 2500 µm^2^ depict shank widths of 100 µm. Additionally, PI and PaC are linked to cross sections of 350 and 700 µm^2^, while Si is linked to 1500 and 2500 µm^2^. Arrows show the main trends. Significant differences in (b) and (c) are shown after performing post-hoc pairwise testing using non-parametric bootstrap t-tests and Bonferroni correction (* for *p* ≤ 0.05, ** for *p* ≤ 0.01 *** for *p* ≤ 0.001).

Following a similar trend as ITA, the lowest count of dead cells was obtained for PI-50 and PaC-50 when using the highest V_in_, producing on average 1.42 ± 1.02 and 0.63 ± 0.72 dead cells in the trauma area, respectively (Fig. 7a). Differences were mostly encountered using PaC-50 with a V_in_ of 162.5 and 187.5 µm/s. The latter condition caused significantly fewer dead cells than Si-100, and most of PI-100 and PaC-100 insertions, as well as lower than PI-50 and PaC-50 with the slower insertion speeds (Supplementary Figure 6a). While no significant difference was established among the materials (Fig. 7b), shank cross-sections with narrower widths yielded significantly lower dead cell counts than 100 µm wide shanks (Fig. 7c). Moreover, a positive effect due to high V_in_ was observed in PI and PaC probes, especially for a shank width of 50 µm (Fig. 7a and Supplementary Figure 6b,c).

### Success rate of intraretinal insertions

**Figure 8:**
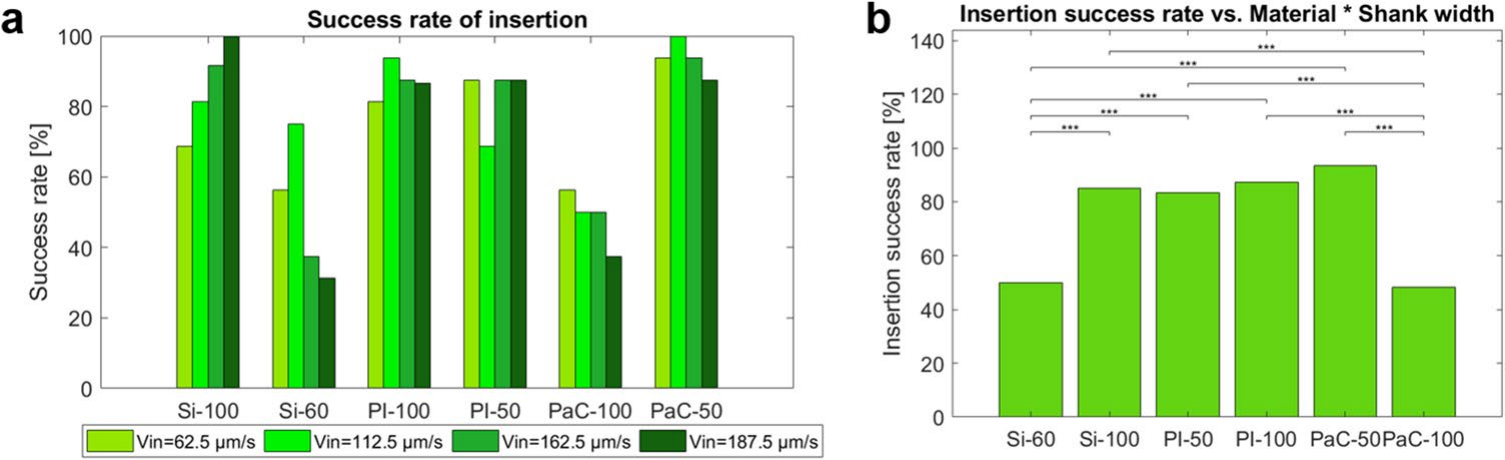
Success rate of intraretinal insertions. The success rate of insertion is shown for individual shanks after 96 insertions (N = 383). (**a**) Overall assessment among each individual probe type (Si-100, Si-60, PI-100, PI-50, PaC-100, and PaC-50) using different insertion speeds V_in_ (62.5, 112.5, 162.5, and 187.5 µm/s). (**b**) Comparisons between the combined properties of material and shank width (50–60 and 100 µm). Significant differences are shown after performing post-hoc pairwise testing using Fisher’s Exact test with Bonferroni correction (* for *p* ≤ 0.05 and *** for *p* ≤ 0.001).

Optical tracking of the experiments confirmed the insertion of intraretinal probes (Supplementary Figure 7), however, aiming for a bidirectional device, the success rate of intraretinal insertions was evaluated by considering only those insertions that yielded recordings of APs in the upper electrodes during the last insertion step as successful. Thus, Si- and flexible- BiMEAs revealed an overall insertion success rate of 87.24%, excluding Si-60 and PaC-100, which yielded a success rate of ~ 50%. While PI probes showed in general a combined success rate that was high (> 85%), the highest success rates were obtained when using Si-100 with a V_in_ of 187.5 µm/s and PaC-50 with a V_in_ of 112.5 µm/s. Likewise, a clear trend of increasing success rate with increasing V_in_ was visible for Si-100 (Fig. 8a).

Additionally, when analysing the success rate by materials, Si and PaC probes showed poor performance, caused by the high failure rate of narrow shanks in the case of Si and wide shanks in the case of PaC. While Si-60 and PaC-100 performed successfully only 50% and 48.44% of the time, respectively, Si-100 and PaC-50 had significant success rates of 85% and 93.33% with respect to Si-60 and PaC-100, respectively (Fig. 8b). The Si-60 case can be explained since a full insertion of the Si shanks was avoided (white arrow in Si-60, Supplementary Figure 7), as the probe carrier, which is directly connected to the shanks, could have amplified the insertion trauma. The latter was observed in preliminary tests, where the direct contact between the carrier and the tissue caused severe neuronal loss, as well as the displacement of the axons of RGCs (Supplementary Figure 8).

In the case of PaC-100, efforts to further insert the shanks into the tissue resulted in collateral attempts to insert the flexible tether holder that separates the shanks from the carrier. However, given the dimensions of the latter structure (800 µm × 200 µm), bending was induced (white arrow in PaC-100, Supplementary Figure 7). Thus, the curvature resulted in retraction of the shanks when further steps were carried out after Z_in_. To avoid bias in the insertion impact evaluations, only Si-60 and Pa-C-100 insertions with clear shank insertion and no holder impact were evaluated.

### Outlook

Continuing the development of bidirectional intraretinal probes^8,9,12^, this work unveiled the development of flexible and penetrating MEAs for retinal applications, for the first time demonstrated the in vitro implementation of flexible intraretinal probes, and revealed the reduced acute trauma induced by the insertion of such devices. The development of flexible BiMEAs embraced strategies reported for intracortical probes, which were adapted to match the requirements of intraretinal applications. Thus, measures like cross-section optimisation, consideration of the anatomical and mechanical features of our target tissue, the use of flexible materials such as polyimide and parylene C, and the establishment of an intraretinal insertion model, allowed us to: (i) tailor the probes to the dimensions of the thin retina, (ii) predict the insertion feasibility of flexible intraretinal probes, and (iii) reduce the biological impact of intraretinal insertions. Even more, the reduction of the effective length of the shanks proved an alternative to the application of an insertion aid to successfully insert the flexible shanks into the neural tissue, an approach that can also benefit the insertion strategies of different neural implants, such as intracortical probes^13,38^.

Moreover, in vitro experiments exhibited successful insertions of flexible BiMEAs into healthy and degenerated mouse retinae. Thus, the spiking activity and the LFPs captured in the recordings confirmed the vitality of the tissue, as well as the access to different retinal layers. These features are essential for a bidirectional strategy that aims to record and stimulate intraretinally. Likewise, the insertion of a probe only 50 µm wide, 7 µm thick, and 145 µm long into a neural tissue so thin as ~ 100 µm (*rd10* retina) was shown for the first time, while still recording the activity at different neural depths.

Furthermore, it was shown that the acute insertion footprint caused by intraretinal insertions was influenced by the combined effect of the probe material, the shank width, (and depending on the latter) by the speed of insertion. Consequently, flexible probes with narrow shanks and high insertion speeds significantly yielded the smallest acute insertion footprints when compared to Si-based devices (see Supplementary Figure 9 for a comparison using the same scale). Given the above and considering the known biocompatibility of polymer-based materials, flexible BiMEAs show the potential for future in vivo intraretinal applications.

## Methods

### Model of intraretinal shanks as columns

Shanks were first classified according to λ^25,26^, which was calculated using Eqs. (1–4),

1 $$ \begin{array}{*{20}c} {\lambda = \frac{Lk}{{r_{x} }}} \\ \end{array} $$

2 $$ \begin{array}{*{20}c} {r_{x} = \left( {\frac{{I_{m} }}{A}} \right)^{\frac{1}{2}} } \\ \end{array} $$

3 $$ \begin{array}{*{20}c} {I_{m} = \frac{{wt^{3} }}{12}} \\ \end{array} $$

4 $$ \begin{array}{*{20}c} {A = wt} \\ \end{array} $$

where L is length of the shank, k is the effective length constant (for a fixed-pinned condition k = 0.7), r_x_ is the minimum radius of gyration, I_m_ is the moment of inertia, A is the cross-sectional area, w is the width, and t is the thickness of the shank. Equations (5) and (6) were used to calculate the theoretical buckling force (P_R_) of the proposed shanks.

5 $$ \begin{array}{*{20}c} {P_{R} = \frac{{\sigma_{c} A}}{{1 + \alpha \left( \lambda \right)^{2} }}} \\ \end{array} $$

6 $$ \begin{array}{*{20}c} {\alpha = \frac{{\sigma_{c} }}{{\pi^{2} E}}} \\ \end{array} $$

Equation 5 corresponds to Rankine-Gordon’s formula, which accounts for the slenderness ratio and the mechanical properties of the column. Equation 6 is a constant that depends on the Young’s modulus (E) and the crushing strength (σ_c_) of the material of the column^25,26^. In this paper, σ_c_ equates to the tensile strength, as it is the maximum stress that a material can withstand before failure. For the calculation of P_R_, E and σ_c_ were 2.76 GPa and 68.95 MPa for PaC (data given by the supplier, Specialty Coating Systems Inc.), 8.45 GPa and 650 MPa for PI^39^, and 170 GPa and 2000 MPa for Si^40^.

### Phantom retina

To mimic the softness of the retina, a PDMS phantom was prepared by mixing the cross-linker and pre-polymer of Sylgard 184 (Dow Corning) with a ratio of 1:45 (w/w). The mixture was degassed in a desiccator under vacuum for 30 min, poured into a metal petri dish with a diameter of 30 mm to achieve a thickness of 7 mm, and cured in an oven at 120 °C for 4 h. A micro-indentation test was used to confirm a compressive elastic modulus of ~ 29 kPa (see Supplementary Figure 10).

### Design and fabrication of flexible intraretinal probes

Based on the design of Si-BiMEAs^9^, flexible BiMEAs are microelectrodes arrays with a Michigan-like design^41^. The probe design comprises four penetrating shanks with an inter-shank distance of 100 µm. Individual shanks have a length of 140/145, 180/185, or 220/255 µm, a width of 50 or 100 µm, a thickness of 7 µm, and a tip angle of 30°. Likewise, each shank has four (for lengths 180–225 µm) or three (for lengths of 140–145 µm) iridium oxide (IrO_x_) electrodes with an interelectrode distance of 12 µm, and a distance from the shank tip to the first electrode of ~ 50 µm. The bottom electrode, intended for electrical stimulation, has a diameter of 25 µm, while the upper electrodes, designed for electrical recording, have a diameter of 15 µm (Polymer-based probes in Fig. 1a, Supplementary Figure 1a). Additionally, the design includes a rectangular tether holder that is 200 µm long and 800 µm wide between the shanks and the contact pad area (Supplementary Figure 1a).

The fabrication of flexible BiMEAs was performed as follows (Supplementary Figure 11): First, a layer of PI or PaC was deposited onto a four-inch Si wafer with a thickness of ~ 3 µm. In a second step, a first metallisation process was performed to pattern a base Ti/Au layer for contact pads, interconnects, and electrodes. Then, a second flexible layer with a thickness of ~ 1 µm was deposited and the interlayer openings corresponding only to the electrode sites were etched. Afterwards, a second metallisation process was carried to sputter a Ti/Pt/IrO_x_/Ti metal stack onto the Ti/Au base that was previously patterned at the electrode sites. A third flexible layer ~ 3 µm thick was then deposited as the encapsulation layer, and a second etching step was performed to subsequently pattern the shape of the probe, open the contact pads and electrode sites, and remove the protective Ti layer on top of the IrO_x_ simultaneously. To finalize the fabrication, the flexible probes were released from the Si wafer and soldered via flip-chip bonding to a 16-channel printed circuit board (PCB) (Supplementary Figure 1b). For a detailed fabrication protocol see Supplementary Methods.

Samples used during the insertion test with the phantom retina were flexible dummy probes that were fabricated with thicknesses of 3, 5, and 7 µm using only the first flexible and metal layer (Supplementary Figure 11a,b).

### Animals

The following mouse strains were used for intraretinal insertions, MEA recordings, and dead cell stainings: C57Bl/6N (in the following referred to as wildtype (WT)) and C57Bl/6J-Pde6b^*rd10*^/J mutants (in the following referred to as *rd10*). WT animals were obtained from Charles River and *rd10* mice were bred locally from breeding pairs obtained from Jackson (strain name: B6. CXB1-*Pde6b*^*rd10*^/J). In this line, the *rd10* mutation was backcrossed onto the C57Bl/6J background for 5 generations before intercrossing to homozygosity. Transgenic mice expressing the genetically encoded Ca^2+^-sensor TN-L15 (gift from Dr. O. Griesbeck, MPI for Neurobiology, Martinsried, Germany) were also taken from the own live-stock breeding at the animal facility at the *Forschungszentrum* Jülich. Animals were kept in a 12-h dark/light cycle with water and food ad libitum. All mouse lines were kept and bred in the animal facility at the *Forschungszentrum* Jülich. All experiments were performed in accordance to the German law for the Protection of Animals and after approval was obtained by the regulatory authorities, the *Forschungszentrum* Jülich (number ICS-4 OE2) and the *Landesamt für Natur*, *Umwelt* und *Verbraucherschutz* of North-Rhine Westfalia.

### Retina preparation

The preparation of retinal samples was carried out at ambient light. The animals were first anesthetised with isoflurane (Actavis Dtl. GmbH &Co. KG) and then killed by decapitation. Immediately, the eyeballs were extracted and immersed in Ames’ medium (Sigma-Aldrich), which was constantly oxygenated with Carbogen gas (95% O_2_, 5% CO_2_—The Linde Group) at a pH of 7.4 at room temperature. The retina was isolated from the eyecup after removal of the cornea, the lens, and the vitreous body. Afterwards, the tissue was cut in halves and stored in the oxygenated medium until it was used. A retinal sample was then mounted into the perfusion chamber, as reported before^9^. With the ganglion cell layer (GCL) facing downwards, the tissue was placed and fixed from the periphery on top of a millipore filter paper (Merck KGaA) that had a central hole of 1.5 mm. Next, the tissue was transferred with the GCL upwards to the perfusion chamber, where it was fixed using insect pins, covered with oxygenated medium, and finally moved to the experimental setup. In this way, a total of four retinal samples were obtained per animal.

### Experimental setup

As previously reported^9^, the experimental setup consisted of a Faraday cage that shielded three main components: (i) a micromanipulator system with three degrees of freedom steered by an SM-6 controller through a LabVIEW interface provided by the manufacturer (Luigs & Neumann), (ii) the headstage of the amplification system for electrical recordings, which in turn had a front-end with a 16-channel PCB that supported the penetrating MEAs during insertion, and (iii) a perfusion chamber, which had a PDMS pillow to support the tissue and permitted a constant flux of oxygenated medium to maintain the vitality of the tissue. Additionally, to allow optical tracking of the insertions, the reservoir of the perfusion chamber was made with a squared quartz glass chamber and a VHX digital microscope (Keyence) supported by a flexible arm was used from outside the cage.

### MEA recordings

The BioMAS, an in-house amplification system^9,42^, was used with a custom-built 16-channel headstage to perform electrical recordings. The sampling rate was set to 20 kHz and a Ag/AgCl pellet (World Precision Instruments) was used as reference electrode. The spiking activity and LFPs were extracted from the band-pass filtered signal (cutting frequencies of 100 Hz and 3 kHz) and low-pass filtered signal (cutting frequency of 100 Hz) of the raw recordings, respectively. Offline data processing was performed using self-written scripts in Matlab 2018 (The MathWorks Inc.) as reported before^9^.

### Intraretinal insertion protocols

Insertion parameters Z_in_, ΔZ, and V_in_ refer to the initial insertion step, the step size of the micro steps after Z_in_, and the insertion speed, respectively.

#### Insertion into a phantom retina

PI and PaC dummy probes with widths of 100 and 50 µm, lengths of 220 and 225 µm, and thicknesses of 3, 5, and 7 µm were inserted stepwise into a PDMS phantom with Z_in_ = ΔZ = 20 µm and a V_in_ set to 62.5 µm/s. After insertion, the shanks were immediately retracted using the same speed and step size.

#### In vitro recordings of WT and *rd10* mouse retinae

PI (50 µm wide, 185 µm long, and 7 µm thick) and PaC (50 µm wide, 185 or 145 µm long, 7 µm thick) BiMEAs were used in in vitro intraretinal insertion experiments in WT and *rd10* retinae, respectively. Z_in_ was set between 40 and 180 µm, ΔZ was between 20 and 40 µm, and V_in_ of 62.5, 162.5, or 185.5 µm/s was employed.

#### Biological impact of intraretinal probes

As reference, Si-based probes that were previously reported for intraretinal applications were used^9^. Thus, Si (100/60 µm wide,1000/312 µm long, 25 µm thick), PI (100/50 µm wide, 180/185 µm long, 7 µm thick), and PaC (100/50 µm wide, 180/185 µm long, 7 µm thick) BiMEAs were tested in intraretinal insertion experiments in vitro in TN-L15 retinae. Four insertions with an inter-insertion distance of ~ 300 µm were performed per retinal sample. Four insertion speeds were tested, setting V_in_ to 62.5, 112.5, 165.5, and 187.5 µm/s for each retinal sample. Insertion steps of 100 µm (Z_in_) were performed until APs were observed at least in the lower electrodes. Z_in_ was repeated 1–2 times in intervals of 10 s if necessary. Further steps with ΔZ between 20 and 40 µm were performed to tune the position of the electrodes within the retinal layers, in such a way that the upper electrodes could record the spiking activity of the retina.

Regarding the in vitro experiments, after positioning the electrodes at the desired depth, the light of the microscope was turned off and the retina was left to recover for 5 min. Afterwards, the vitality of the tissue was checked using a high photopic light stimulus (7.96 µW/mm^2^) with a duration of 500 ms, which was repeated 5 times every 15 s. Retraction of the shanks was then performed with ΔZ of 20 µm every 5–10 s.

### Dead stainings

An assessment of the dead cells originating after the acute intraretinal insertions was performed with a dead cell staining. To distinguish RGCs, TN-L15 retinae with a strong visible fluorescence of RGCs were used. After the retraction of the shanks, the retinal samples were stored in oxygenated Ames’ medium until the insertions of all samples were completed. Based on protocols reported to stain live tissue slices ex vivo^43^, retinae were processed as follows: For four retinal samples, the staining solution was prepared by mixing 3 mL of fresh oxygenated Ames’ medium with 15 µL of 2 mM Ethidium Homodimer-1 (ThermoFischer). The retinal samples were moved into a 24-well plate, where four wells were each previously filled with 700 µL of the staining solution. Then, the well plate was placed inside a closed wet chamber that was properly oxygenated. Next, the constantly oxygenated wet chamber was placed on an orbital shaker and the tissues were stained for 20 min at 60 rpm. To remove the dye, the samples were moved into a well filled with fresh medium and shaken for 10 min. Finally, the tissue pieces were moved into a well with new medium and stored until imaging.

### Confocal laser scanning microscopy

Fluorescent retinal wholemounts of TN-L15 mice were covered with oxygenated Ames’ medium and analysed with a confocal laser scanning microscope (TCS SP5 II, Leica Microsystems). Using 10 × and 20 × (NA 0.7) objectives, optical sections were made at an image resolution of 1024 × 1024 pixels. Z-stacks were taken in different focal planes with a step size of 5–9 µm and a total thickness of 60–150 μm.

### Image processing

To perform an assessment of the acute trauma generated by the insertion of intraretinal probes, Z-stacks were processed using ImageJ^44^. Maximum intensity projections of individual and merged channels were obtained first (red for dead cells and green for RGCs). Then, the areas affected by the insertion of each individual shank were manually outlined, classified as regions of interest (ROIs), and the corresponding areas were calculated. Each ROI corresponds to ITA. The borders of ITA were identified according to the visible neuronal loss (insertion holes) present in the green channel and the dead cells within and around the area. Maximum intensity images of the red channel were thresholded manually and converted into binary images. To remove isolated pixels and separate merged particles, the binary “open” operation was applied followed by a watershed segmentation. Further counting of dead cells was carried out within each ROI using the automatic function “Analyse Particles” in ImageJ. In this way, for each shank insertion the following data was extracted: the insertion trauma area (ITA), the insertion trauma area ratio (ITR), defined as the ratio between ITA and the cross-section of the shank, and the count of dead cells (# dead cells).

### Statistical analysis

Statistical analysis was performed in Matlab 2018 (The MathWorks Inc.) using the “Statistics and Machine Learning” toolbox together with self-written scripts. Considering that the normality of the studied variables ITA, ITR, and # dead cells, was rejected after performing Lilliefors test; non-parametric bootstrap tests with a pooled resampling method were applied^45^. Accordingly, comparisons for the interaction effects of material, cross-section or shank width, and insertion speed were performed with post-hoc pairwise testing using non-parametric bootstrap t-tests with ten thousand bootstrap replicates and Bonferroni correction. Additionally, comparisons regarding the success rate of insertion were made with post-hoc pairwise testing using Fisher’s Exact test with Bonferroni correction.

## Supplementary information

Supplementary Information.

Supplementary Information.
