# Supplementary material for: Development and in vitro validation of flexible intraretinal probes
Source: Sci Rep. 2020 Nov 16;10:19836. doi: 10.1038/s41598-020-76582-5 (PMC7669900; doi:10.1038/s41598-020-76582-5)
Supplement: Supplementary file 1 — Supplementary Information. [file 41598_2020_76582_MOESM1_ESM.pdf]

Supplementary Information

Development and *in vitro* validation of flexible intraretinal probes

V. Rincón Montes[1,5], J. Gehlen[2], S. Ingebrandt[3], W. Mokwa[3], P. Walter[4], F. Müller[2], and A. Offenhäusser[1,5*]

[1]Bioelectronics, Institute of Biological Information Processing-3, Forschungszentrum Jülich, Jülich, Germany

[2]Molecular and Cellular Physiology, Institute of Biological Information Processing-1, Forschungszentrum Jülich, Jülich, Germany

[3]Institute of Materials in Electrical Engineering 1, RWTH Aachen University, Aachen, Germany

[4]Department of Ophthalmology, RWTH Aachen University, Aachen, Germany

[5]RWTH Aachen University, Aachen, Germany

[*] a.offenhaeusser@fz-juelich.de

Supplementary Methods

Fabrication protocol of flexible BiMEAs

The fabrication of flexible BiMEAs was mainly conducted at the HNF cleanroom facility at Forschungszentrum Jülich.

First, a flexible layer with a thickness of ~3 µm was deposited onto a four-inch Si wafer (Supplementary Figure 10a). In a second step, a first metallisation process was performed. Here, a metal base layer for contact pads, interconnects, and electrodes was patterned after spin-coating a stack of LOR3B (MicroChem Corp.) and nLOF2020 (MicroChemicals GmbH) photoresist at 3000 rpm onto the flexible layer. The wafer was then exposed to 40 mJ/cm^2 of UV light, developed for 35 s in AZ 326 MIF (MicroChemicals GmbH), and evaporated with a metal stack of 10/100 nm of Ti/Au (Supplementary Figure 10b). Lift-off of the metal was performed under acetone for at least 2h and LOR3B residues were removed in AZ 326 MIF for ~5 min. Then, a second flexible layer with a thickness of ~ 1 µm was deposited (Supplementary Figure 10c). Subsequently, a photolithography and a reactive ion etching (RIE) step was performed to remove the interlayer openings at the electrode sites (Supplementary Figure 10d). To this end, an etching mask was patterned on top of the interlayer by spin-coating AZ 9260 photoresist (MicroChemicals GmbH) at 3000 rpm, exposing it to 900 mJ/cm^2 of UV, and developing for ~ 7.5 min in AZ 326 MIF. The latter was followed by a RIE step using an O_2/CF_4 gas mixture (36/4 sccm) with RF/ICP powers of 50/500 W and a process pressure of 0.007 mbar, yielding an etching rate of ~ 800 and 600 nm/min for PaC and PI, respectively.

As shown in Supplementary Figure 10e, a second metallisation step was performed to coat the Ti/Au electrodes with IrO_x. Thus, a deposition mask of nLOF2020/LOR3B at the electrode openings was patterned. This time the resists were spin-coated at 2000 rpm to ensure a total resist thickness of ~ 2.5 µm. Afterwards, a stack of Ti/Pt/IrO_x/Ti with a thickness of 10/100/250/10 nm was sputtered (performed at the cleanroom facility at IWE-1, RWTH

Aachen University), and a lift-off process was carried out as described above. Afterwards, a third flexible layer of ~ 3 µm was deposited, which was used as the passivation layer (Supplementary Figure 10f). To pattern the shape and expose the openings of the contact pads and electrodes, a second photolithography and RIE step was performed (Supplementary Figure 10g). Considering that the total thickness of the polymers was ~ 7 µm, an etch mask with a thickness of ~ 20 µm was patterned. To this end, two stacked layers of AZ 9260 photoresist spin-coated at 2400 and 2100 rpm each were patterned after a UV exposure of 2100 mJ/cm^2 using a development time of ~20 min in AZ 326 MIF. A RIE step as described before was carried out with an additional etching step to remove the Ti layer at the top of the electrodes, which served as a protective layer after the long etching process to pattern the polymers (etching times of ~ 8-12 min). In all RIE steps, resist residues corresponding to the etch mask were stripped using AZ-100 remover (MicroChemicals GmbH) in an ultrasound bath for at least 10 min. Then, the samples were rinsed 3 times with fresh isopropanol. Finally, individual flexible probes were released from the Si wafers. In the case of PaC probes, a drop of water was used to facilitate the release with tweezers. As for the PI samples, a Cr-etchant solution (Sigma-Aldrich) was used for ~ 40 min. Individual probes were then rinsed 3 times in fresh Milli-Q water to remove any Cr-etchant residues from the probes.

Deposition of the flexible polymers was performed as follows. In the case of PaC, 6 g (3 µm) and 1g (1 µm) of PaC dimer were used to coat the wafers via chemical vapor deposition (CVD) using a PDS 2010 coater (Specialty Coating Systems Inc.), The adhesion promoter silane A-174 (Specialty Coating Systems Inc.) was applied through a chamber swipe method for the deposition of the second and third PaC layers. As for PI, a process similar to that reported previously was followed[1]. First, a sacrificial layer with 10/100/50 nm of Cr/Au/Cr was evaporated onto a blank Si wafer prior to the deposition of the first flexible layer. Then, each PI layer was achieved by spin-coating first the adhesion promoter VM-652 (HD Microsystems) at 3000 rpm, followed by a layer of PI-2610 (HD Microsystems), which was immediately spin-coated at 2000 or 5000 rpm to achieve ~3 µm or ~1 µm, respectively. Afterwards, the PI layer was soft-baked at 120°C for 4 min with a slow ramp on a proximity hot plate and subsequently cured using a convection oven in a nitrogen environment with a temperature ramp of 4°C/min until 200°C, a second ramp of 2.5°C/min until 350°C, holding the temperature for 30 min, and cooling down with a ramp of 2.5°C/min until room temperature. After this curing step, the PI layers were ready for further processing.

Supplementary Figures

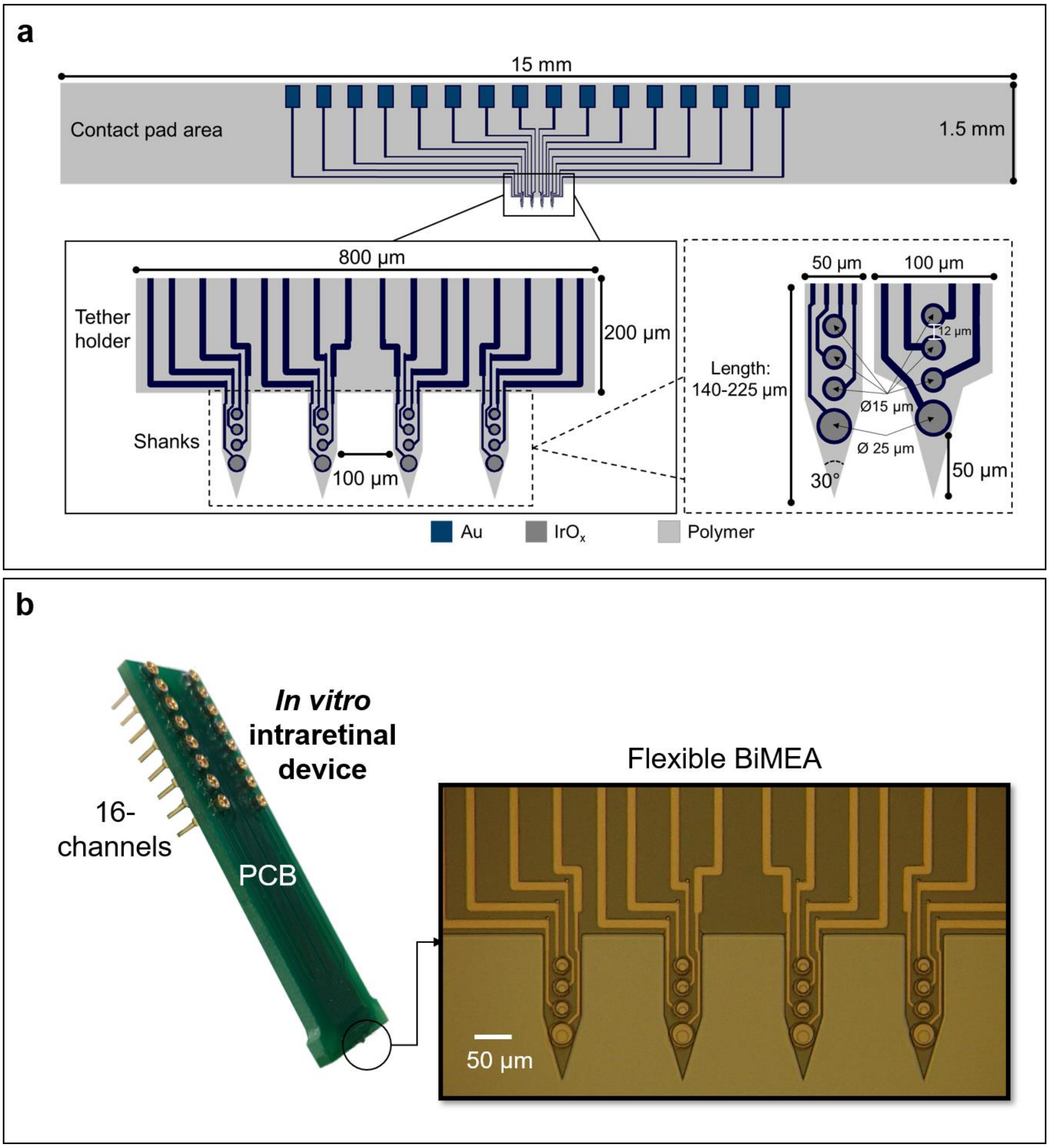

Figure 1. ***In vitro*** **intraretinal devices.** (a) Design of Flexible BiMEAs consist of a contact pad area, a tether holder, and four penetrating shanks, forming an electrode array of 3 (140/145 µm long shank, not shown) or 4 electrode (180-225 µm long shank) sites per shaft. (b) An *in vitro* intraretinal device comprises a printed circuit board (PCB) with 16 channels, and a flexible BiMEA.

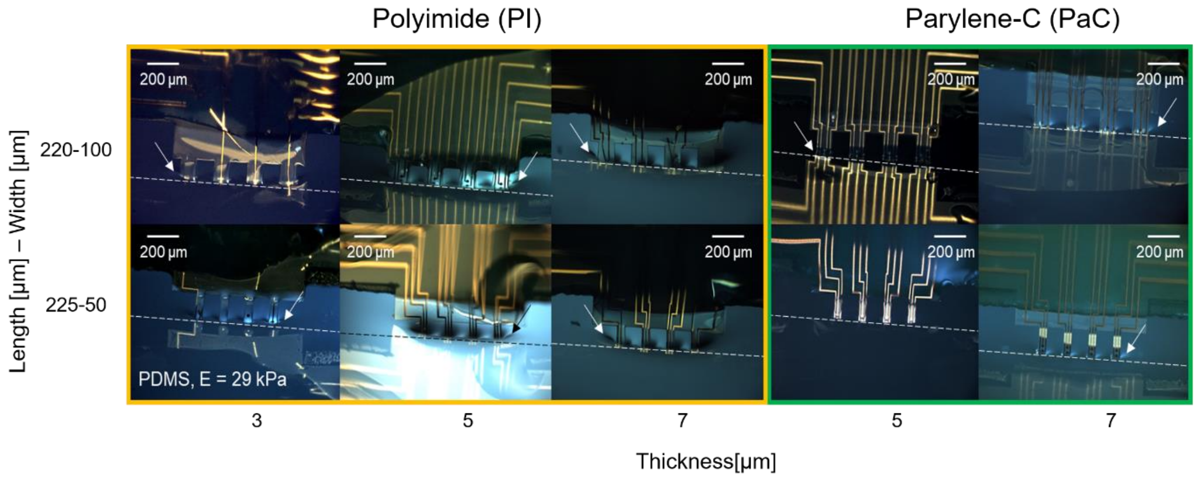

Figure 2. Insertion test into phantom retina. Insertion test of flexible intraretinal probes into a polydimethylsiloxane (PDMS) phantom retina with a Youngs' modulus (E) of 29 kPa for polyimide (yellow square) and parylene-C (green square) probes. Dashed lines exhibit the phantom border and arrows indicate the presence of dimpling.

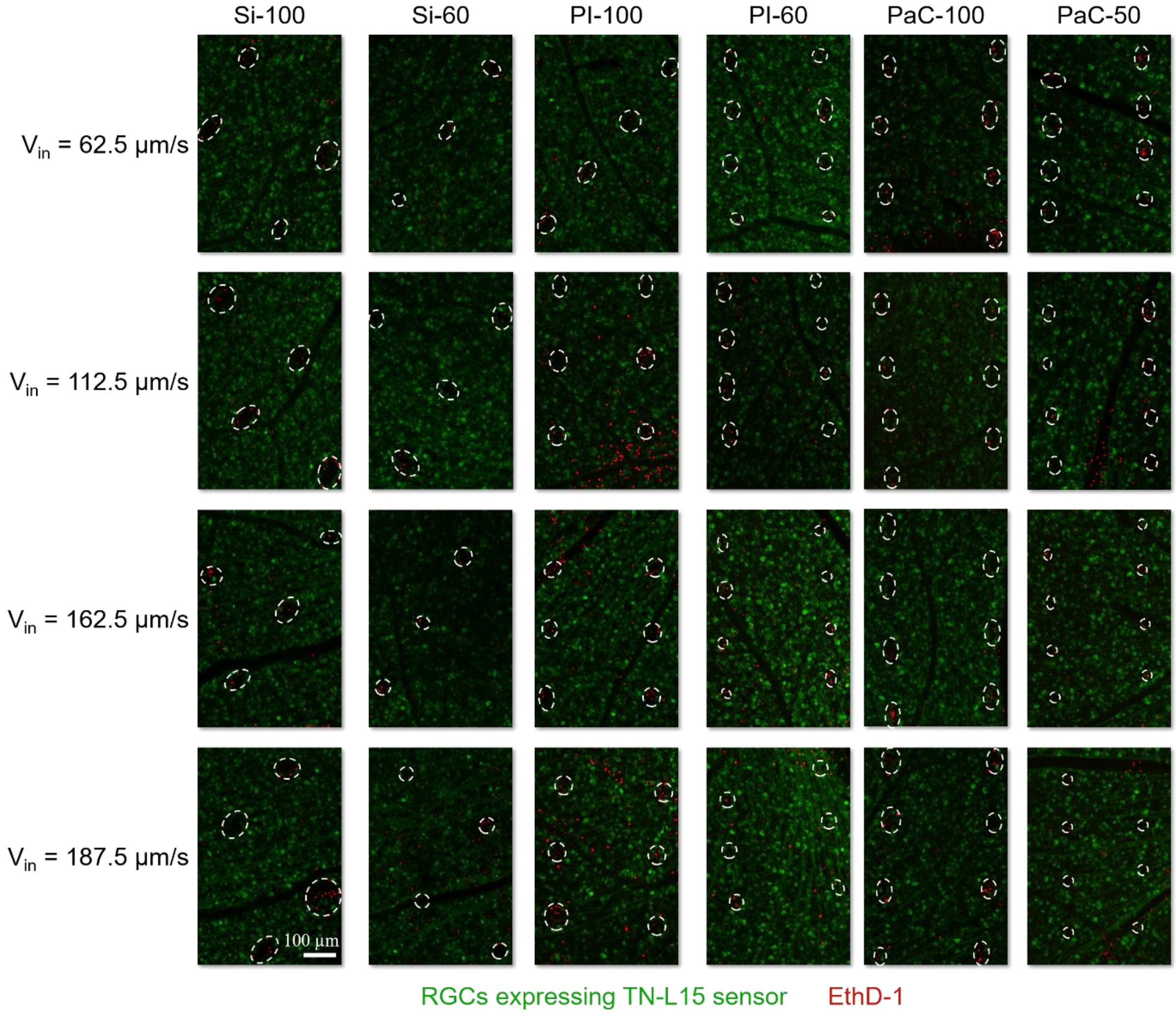

Figure 3. Dead cell stainings after intraretinal insertions in TN-L15 mouse retinae. Ethidium homodimer (EthD-1) was used to stain dead cells (red) in TN-L15 retinae containing RGCs expressing the Ca^{2+} sensor TN-L15 (green). The images show the top view of the maximum intensity projection of the stainings. Six different probes were tested (columns) using different insertion speeds V_{in} (rows). The code at the top of each column corresponds to the material and shank width of each device. White dashed lines enclose the insertion trauma area (ITA).

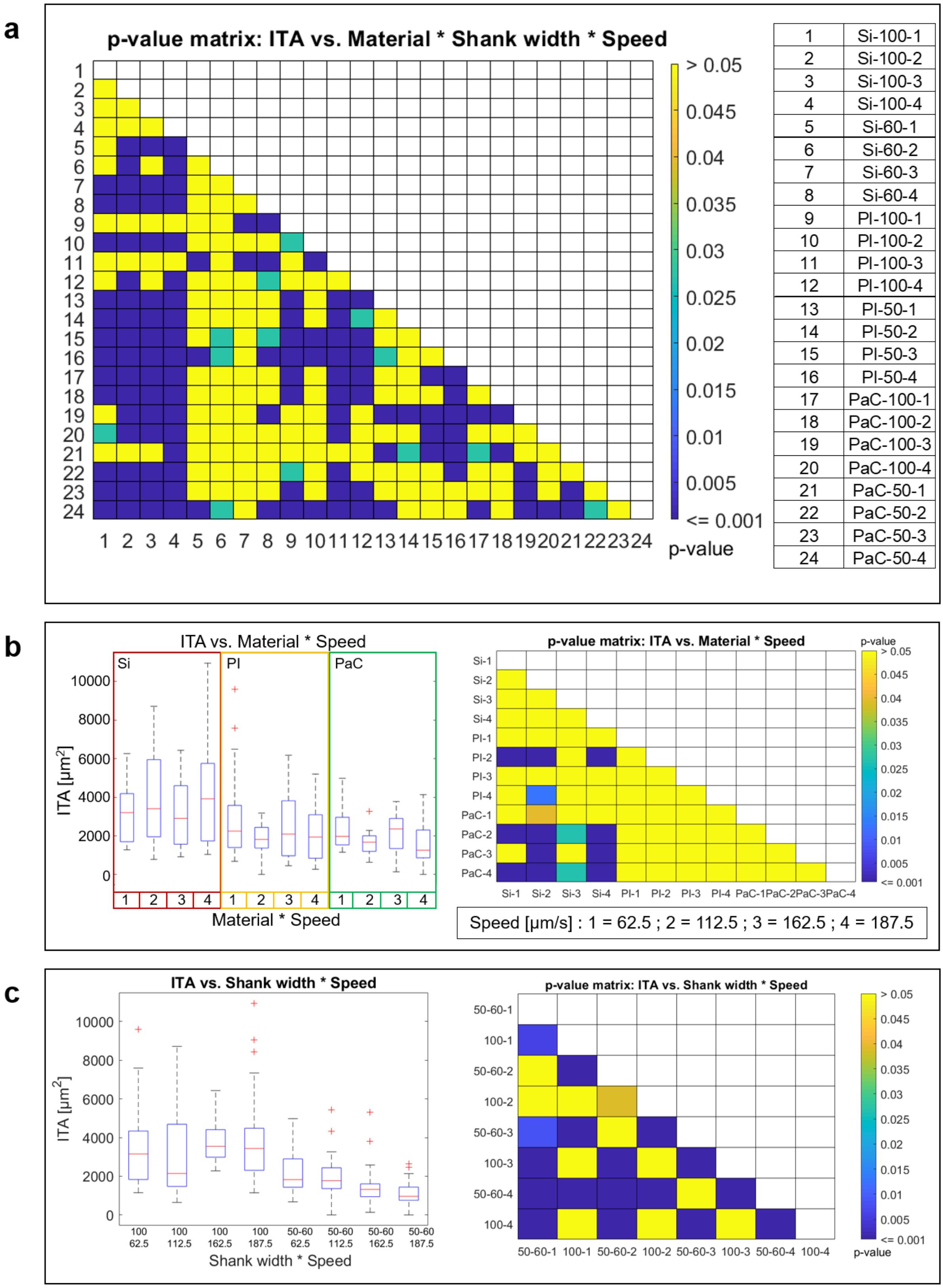

Figure 4. Material, shank width, and insertion speed effects on ITA of intraretinal probes. (a) P-value matrix of non-parametric pairwise comparisons for the interaction effect of material, shank width, and insertion speed. The tested probes are coded according to Material – Shank width – Insertion speed (table at the right). In turn, each insertion speed is coded as: 1 = 62.5 µm/s, 2 = 112.5 µm/s, 3 = 162.5 µm/s, and 4 = 187.5 µm/s. Grouped boxplots and the corresponding p-value matrix for the combined effect of (b) material and speed and (c) shank width and speed. Speed is coded as in (b). P-values were obtained after performing post-hoc pairwise testing using non-parametric bootstrap t-tests and Bonferroni correction. P-values greater than 0.05 are coloured light yellow and show no significance, and p-values below 0.05 denote statistical significance and follow the colour code of the colour bar at the right.

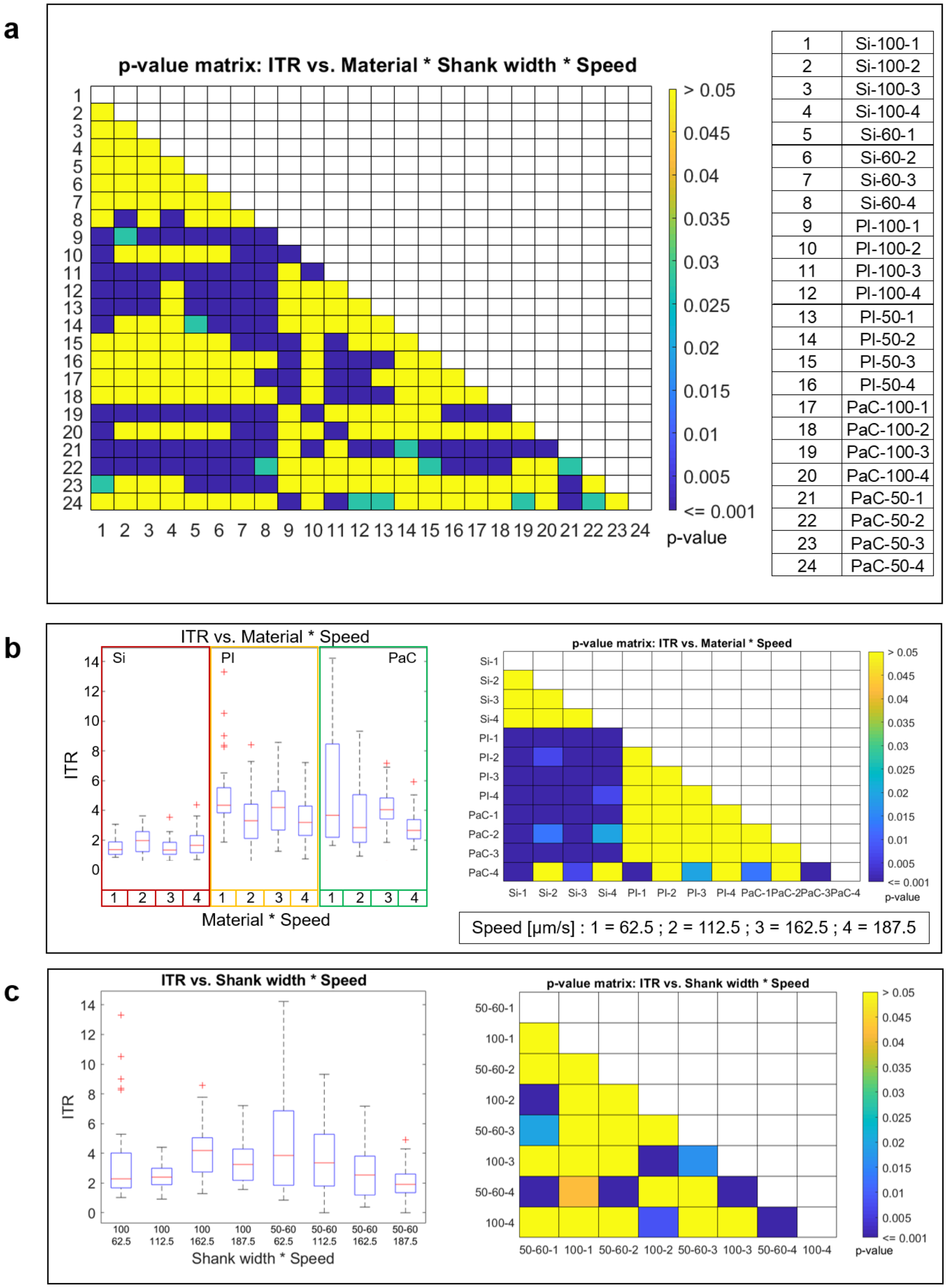

Figure 5. Material, shank width, and insertion speed effects on ITR of intraretinal probes. (a) P-value matrix of non-parametric pairwise comparisons for the interaction effect of material, shank width, and insertion speed. The tested probes are coded according to Material – Shank width – Insertion speed (table at the right). In turn, each insertion speed is coded as: 1 = 62.5 µm/s, 2 = 112.5 µm/s, 3 = 162.5 µm/s, and 4 = 187.5 µm/s. Grouped boxplots and the corresponding p-value matrix for the combined effect of (b) material and speed and (c) shank width and speed. Speed is coded as in (b). P-values were obtained after performing post-hoc pairwise testing using non-parametric bootstrap t-tests and Bonferroni correction. P-values greater than 0.05 are coloured light yellow and show no significance, and p-values below 0.05 denote statistical significance and follow the colour code of the colour bar at the right.

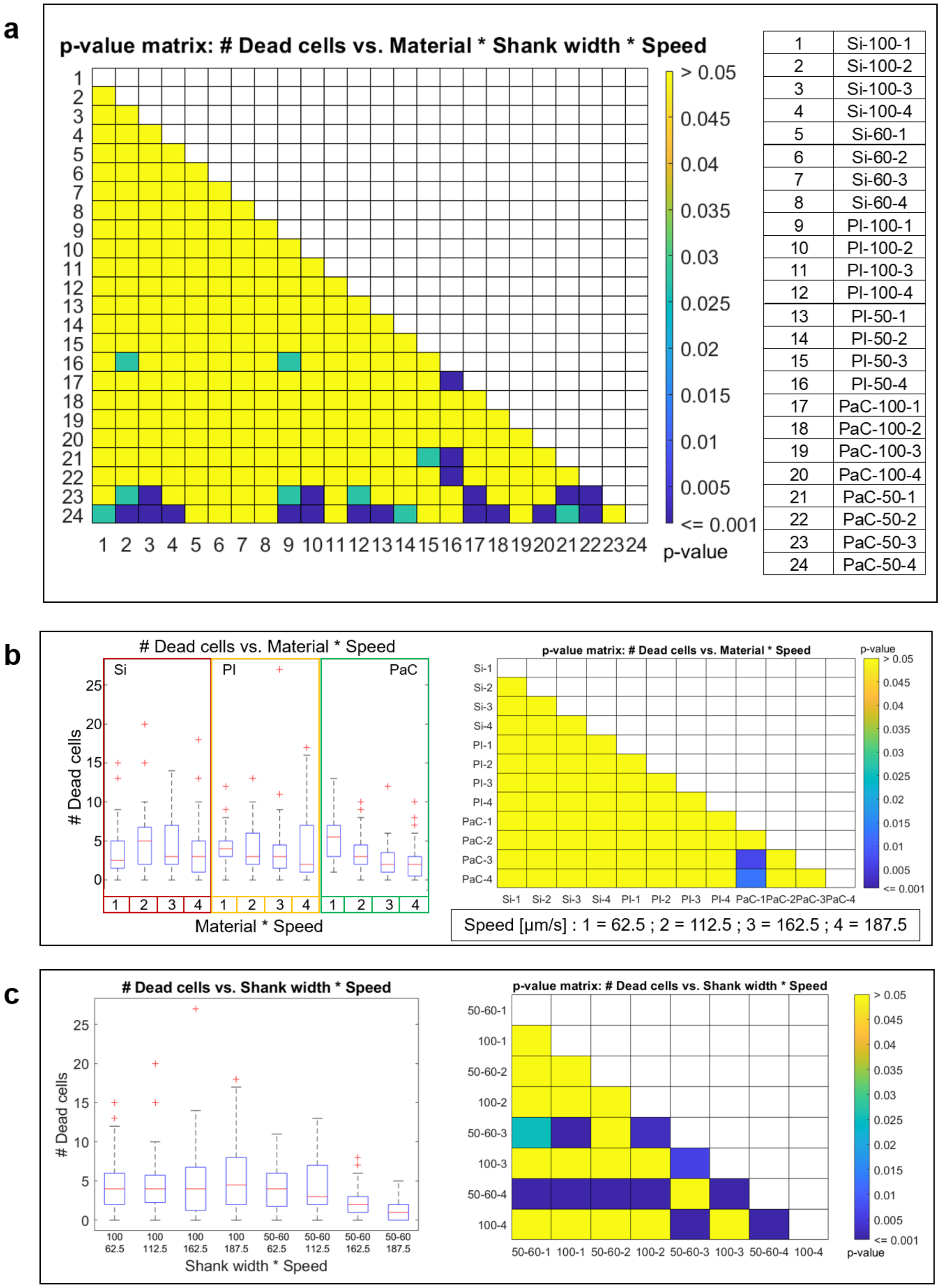

Figure 6. Material, shank width, and insertion speed effects on the count of dead cells after an intraretinal insertion. (a) P-value matrix of non-parametric pairwise comparisons for the interaction effect of material, shank width, and insertion speed. The tested probes are coded according to Material – Shank width – Insertion speed (table at the right). In turn, each insertion speed is coded as: 1 = 62.5 µm/s, 2 = 112.5 µm/s, 3 = 162.5 µm/s, and 4 = 187.5 µm/s. Grouped boxplots and the corresponding p-value matrix for the combined effect of (b) material and speed and (c) shank width and speed. Speed is coded as in (b). P-values were obtained after performing post-hoc pairwise testing using non-parametric bootstrap t-tests and Bonferroni correction. P-values greater than 0.05 are coloured light yellow and show no significance, and p-values below 0.05 denote statistical significance and follow the colour code of the colour bar at the right.

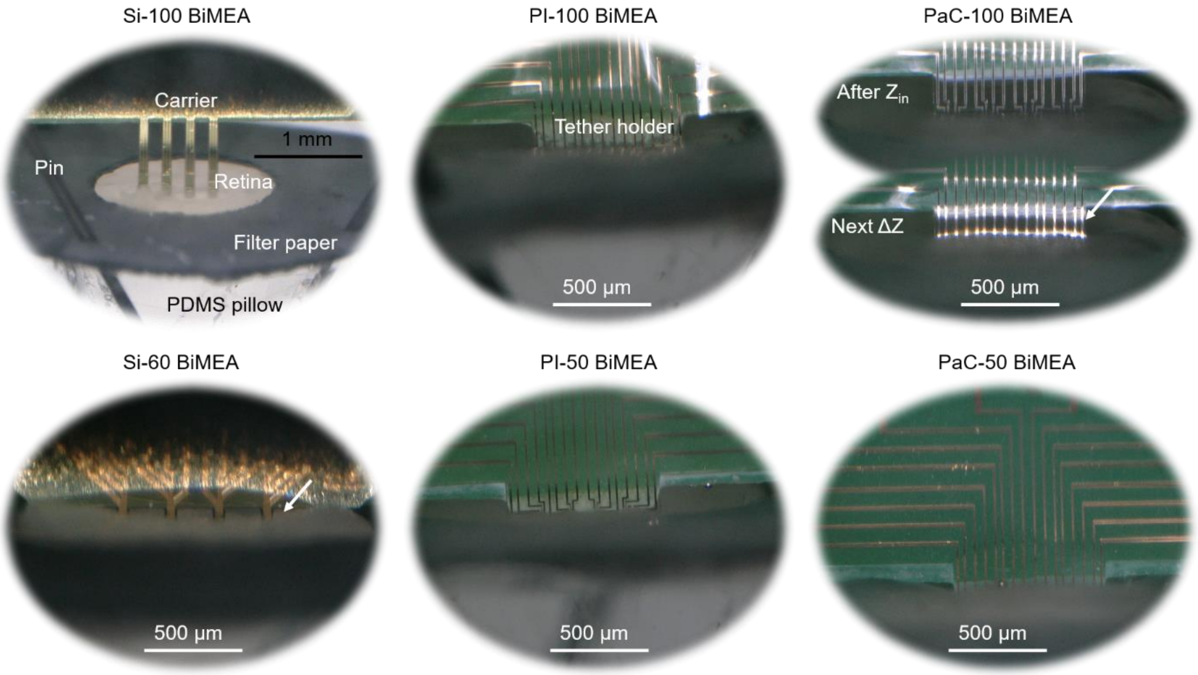

Figure 7. Optical follow-up of intraretinal insertions. Photos of typical insertions with Si-based BiMEAs (Si-100 and Si-60), with PI-based BiMEAs (PI-100 and PI-50), and PaC-based BiMEAs (PaC-100 and PaC-50). For all cases, the retina was carried by a filter paper and placed on top of a PDMS pillow with the ganglion cell layer facing up. Si shanks were held directly by a stiff carrier, while PI and PaC shanks had a flexible tether holder to avoid the direct contact between the shanks and the stiff carrier. The white arrow in Si-60 points out the non-inserted area of the shanks directly connected to the carrier of the probe. The white arrow in PaC-100 shows the bending of the tether holder that supports the flexible shanks.

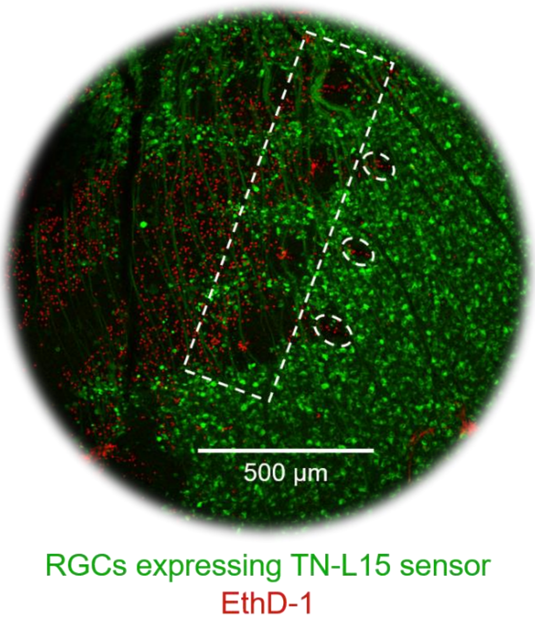

Figure 8. Preliminary acute insertion of a Si-60 probe. Dead cell staining of a wildtype TN-L15 mouse retina after the insertion of a Si-60 probe. Ethidium homodimer (EthD-1) was used to stain dead cells in red and retinal ganglion cells expressing the Ca^{2+} sensor TN-L15 are visible in green. The dashed white rectangle frames the extended trauma area after fully inserting the Si shanks. The direct contact of the carrier of the shanks with the tissue caused more neuronal loss due to bigger insertion holes and displacement of the axons of retinal ganglion cells. As comparison, the white dashed circles enclose the insertion trauma area of a previous insertion where the shanks were not fully inserted.

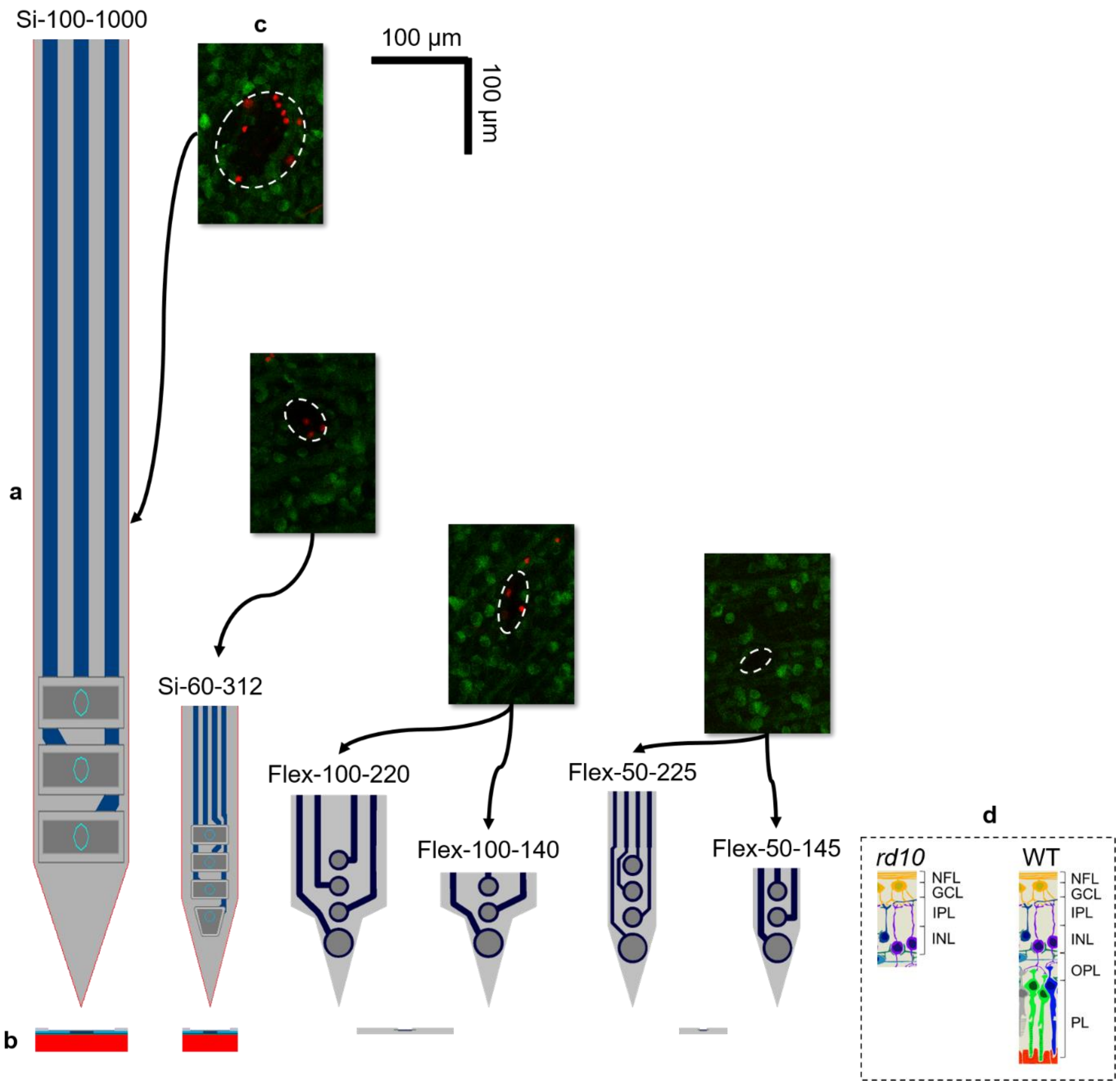

Figure 9. Shank dimension and acute insertion footprint comparison. a) Front and b) cross-section view of penetrating shanks for intraretinal applications. Shanks are coded by material-width-length. Si stands for silicon, Flex for flexible material, such as PI or PaC. c) Top view of dead cell stainings in TN-L15 retinae for four types of probes. In green, RGCs expressing the TN-L15 sensor. In red, dead cells stained with ethidium homodimer. The acute insertion trauma area, also referred as footprint, is enclosed with white dashed lines. The stainings shown for Flex-100 and Flex-50 correspond to a PI-100 and a PaC-50 probes, respectively. d) Schematics of a coronal section of a degenerated (*rd10*) and a healthy (WT) retina. All images are in the same scale of the scale bars (100 µm). Intraretinal layers are coded as: NFL = nerve fibre layer, GCL = ganglion cell layer, IPL = inner plexiform layer, INL = inner nuclear layer, OPL = outer plexiform layer, and PL = photoreceptor layer.

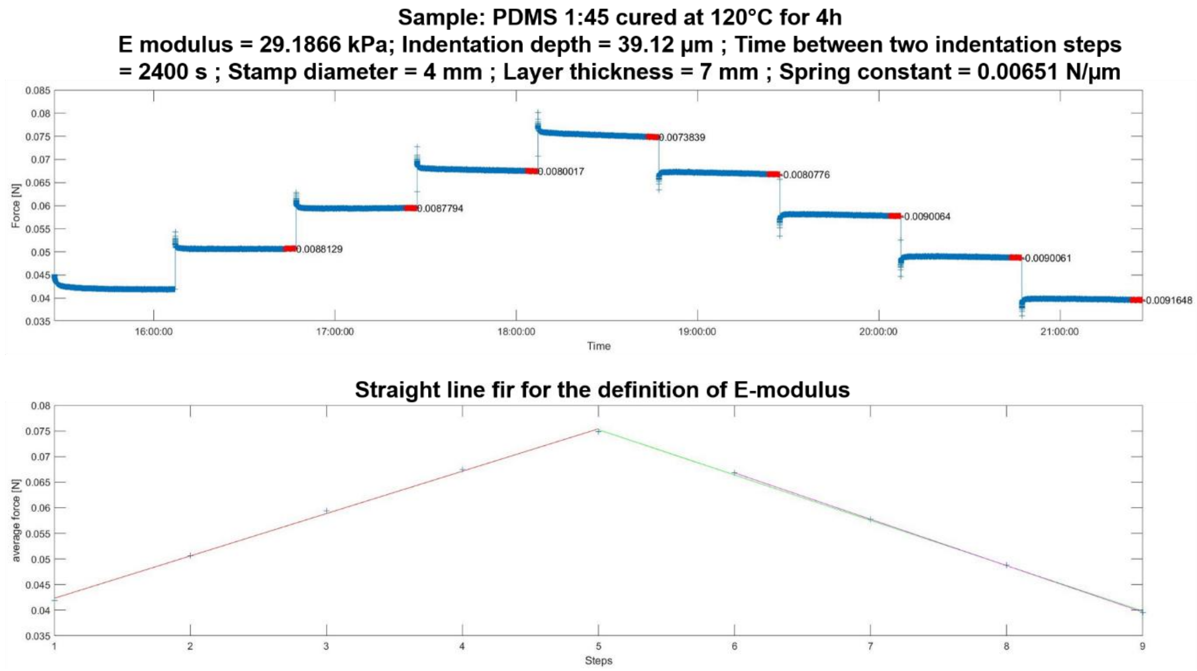

Figure 10. Micro-indentation test results of phantom retina. The Young's modulus (E) was estimated for a phantom PDMS retina (1:45 mixing ratio of cross-linker: pre-polymer). The test was performed using the experimental setup of the Institute of Biological Information Processing-2 (IBI-2) at Forschungszentrum Jülich, which is based on the indentation setup reported by Levental et al., 2010[2].

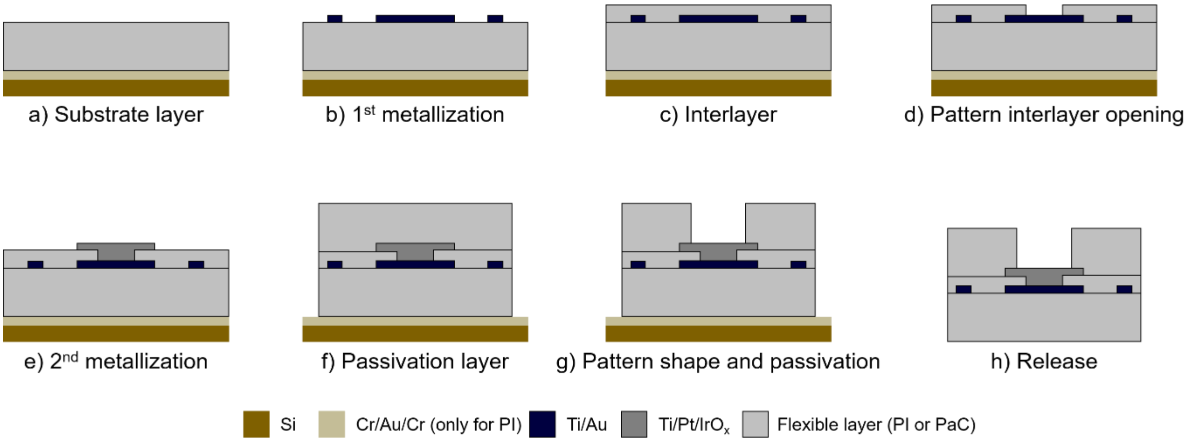

Figure 11. Fabrication flow of flexible BiMEAs. a) A first flexible layer (substrate layer) was deposited via spin-coating for PI or chemical vapor deposition (CVD) for PaC. b) A first metal layer of Ti/Au was patterned on the first flexible layer. c) A second flexible layer (interlayer) was deposited to insulate the first metal layer. d) The interlayer was removed at the electrode sites (interlayer openings). e) A second metallisation process was performed to pattern a Ti/Pt/IrO_x/Ti coating at the electrode sites. f) A third flexible layer was deposited as the passivation layer. g) The flexible layers were etched at the contact pad and electrode sites, along with the shape of the final probe. The last Ti layer was also etched at this step. h) The flexible probes were released from the Si wafer using a drop of water and tweezers for PaC or Cr-etchant solution and Milli-Q rinsing baths for PI.

Supplementary Tables

Table 1. Cross-sectional footprint comparison among substrate materials. The footprint, corresponding to the insertion trauma area (ITA), and the insertion trauma area ratio (ITR) after acute and chronic implantation of penetrating neural probes were estimated for silicon (Si), polyimide (PI), and parylene-C (PaC) probes from the given references.

Probe material	Dimensions [µm] (thickness x width) or (diameter)	Cross-section [µm²]	Footprint type	Bare footprint [µm²]	ITR	Reference
Si	15x120	1,800	Acute	2,538.81	1.41	Szarowski et al., 2003[3]
Si	200	31,416	Chronic (6-12 weeks)	-	1.33-2.1	Thelin et al., 2011[4]
Si	50	1,963	Chronic (6-12 weeks)	-	1-1.89	Thelin et al., 2011[4]
Si	15x250	3,750	Chronic (4-8 weeks)	3,708.82	0.99	Lee et al., 2017[5]
**Si**	**25x100**	**2,500**	**Acute**	**4,064.93**	**1.63**	**This work**
**Si**	**25x60**	**1,500**	**Acute**	**1,757.63**	**1.17**	**This work**
PI	21.3x250	5,325	Chronic (4-8 weeks)	7,808.64	1.47	Lee et al., 2017[5]
**PI**	**7x100**	**700**	**Acute**	**2,095.49**	**2.91**	**This work**
**PI**	**7x50**	**350**	**Acute**	**782.17**	**2.17**	**This work**
PaC	11x300	3,300	Chronic (28 days)	44,070.91	13.35	Kim et al., 2013[6]
PaC	20x35	700	Chronic (6 months)	38,888.12	55.55	Sohal et al., 2014[7]
PaC	8x36	288	Chronic (6 weeks)	3,973.83-9,488.54	13.8-32.9	Wu et al., 2015[8]
**PaC**	**7x100**	**700**	**Acute**	**1,496.61**	**2.14**	**This work**
**PaC**	**7x50**	**350**	**Acute**	**861.71**	**2.46**	**This work**

Supplementary videos

Supplementary video 1: *In vitro* insertion of a flexible intraretinal probe into a wildtype mouse retina using an insertion speed of 162.5 µm/s. A parylene-C-BiMEA with a shank width of 50 µm, a length on 185 µm, and a thickness of 7 µm was used.

Supplementary References

1. Kireev, D., Rincón Montes, V., Stevanovic, J., Srikantharajah, K. & Offenhäusser, A. N 3 -MEA Probes : Scooping Neuronal Networks. *Front. Neurosci.* **13**, 1–10 (2019).

2. Levental, I. *et al.* A simple indentation device for measuring micrometer-scale tissue stiffness. *J. Phys. Condens. Matter* **22**, 1–9 (2010).

3. Szarowski, D. H. *et al.* Brain responses to micro-machined silicon devices. *Brain Res.* **983**, 23–35 (2003).

4. Thelin, J. *et al.* Implant size and fixation mode strongly influence tissue reactions in the CNS. *PLoS One* **6**, 1–10 (2011).

5. Lee, H. C. *et al.* Histological evaluation of flexible neural implants; Flexibility limit for reducing the tissue response? *J. Neural Eng.* **14**, 1–12 (2017).

6. Kim, B. J. *et al.* 3D Parylene sheath neural probe for chronic recordings. *J. Neural Eng.* **10**, 1–16 (2013).

7. Sohal, H. S. *et al.* The sinusoidal probe: A new approach to improve electrode longevity. *Front. Neuroeng.* **7**, 1–14 (2014).

8. Wu, F. *et al.* Silk-backed structural optimization of high-density flexible intracortical neural probes. *J. Microelectromechanical Syst.* **24**, 62–69 (2015).
